# Prevalence, Risk Factors and Impacts Related to Mould-Affected Housing: An Australian Integrative Review

**DOI:** 10.3390/ijerph19031854

**Published:** 2022-02-07

**Authors:** Lisa Coulburn, Wendy Miller

**Affiliations:** School of Architecture and Built Environment, Faculty of Engineering, Queensland University of Technology, Brisbane, QLD 4000, Australia; w2.miller@qut.edu.au

**Keywords:** building characteristics, health, housing conditions, mold, mould, risk factors, Australia

## Abstract

In response to an Australian governmental inquiry into biotoxin-related illness, the purpose of this integrative review is to bring together the current state of evidence on the prevalence, risk factors and impacts related to mould-affected housing in an Australian context, in order to inform building, housing and health research, practice and policy. The robust integrative review methodology simultaneously sought quantitative and qualitative studies and grey literature from multiple disciplines, identifying only 45 studies directly relating to Australian housing and indoor mould. Twenty-one studies highlight negative health impacts relating to indoor residential mould, with asthma, respiratory, allergy conditions and emerging health concerns for chronic multiple-symptom presentation. The majority of studies reported risk factors for indoor mould including poor housing conditions, poor-quality rental accommodation, socioeconomic circumstance, age-related housing issues and concerns for surface/interstitial condensation and building defects in newer housing. Risks for indoor mould in both older and newer housing raise concerns for the extent of the problem of indoor mould in Australia. Understanding the national prevalence of housing risks and “root cause” associated with indoor mould is not conclusive from the limited existing evidence. Synthesis of this evidence reveals a lack of coverage on: (1) national and geographical representation, (2) climatical coverage, (3) housing typologies, (4) housing defects, (5) maintenance, (6) impact from urbanisation, and (7) occupant’s behaviour. This integrative review was key in identifying emerging housing and health concerns, highlighting gaps in data and implications to be addressed by researchers, practice and policy and acts as a comprehensive holistic review process that can be applied to other countries.

## 1. Introduction

Indoor dampness resulting in mould-affected housing was previously estimated to affect 10–50% of dwellings in Australia, Europe, North America, India and Japan [1] with an increase in prevalence when located close to bodies of water, coastal areas or previously flood-affected locations [2]. Mould-affected housing is reported in both developed and developing countries, urban and rural locations, and across all housing types and climate zones.

In recent prevalence studies, dampness and indoor mould indicators affect up to 21% of European homes [3,4], up to 27% of homes in Northern Europe [5,6], up to 47% of American homes [7,8] and 12–78% of New Zealand homes [9,10,11]. In urban, suburban and rural China, up to 12% of housing has indoor mould and up to 55% of homes are affected by window condensation [12,13,14]. A worldwide study of both affluent (high-income) and non-affluent countries reported that up to 47% of homes have the presence of damp or indoor mould [15]. Although few studies exist in tropical regions, a recent study in southern India reported that 50% of homes had dampness problems [16] and a study in northern Thailand reported on indoor mould present in both the “dry” and “wet” seasons in 7.1% of homes and water leakage affecting 28.2% of homes [17]. Despite improvements in building regulations and housing codes in developed countries [18,19,20] and our increased understanding of key building, socioeconomic and occupancy risk factors for indoor mould [1], mould-affected housing is still a persistent and common problem worldwide.

Indoor residential mould is the result of either singular impacts, such as weather events and flooding, or the synergistic effects of interrelated factors, multiple housing condition problems or socioeconomic situations [1,21,22]. These interrelated factors intersect multiple research disciplines and industry domains, and may include the synergistic effects of building failure, poor construction, housing maintenance, occupant behaviours, socioeconomic factors, fuel poverty, regulatory factors, location, climate factors, climate adaption, globalisation, urbanisation and climate adaption [1,22,23,24,25]. An increase in the occurrence of extreme weather events such as cyclones, hailstorms, storm surge, flooding and temperature extremes has both current and future implications for an increased risk of water-damaged housing and indoor mould in climate-sensitive countries, flood-sensitive locations and coastal communities [26,27,28].

Indoor mould growth occurs when sufficient moisture is available. Prolonged exposure to moisture within a building can impact the function of insulation and increase energy use and costs [29], shorten the life of building materials and eventually lead to high replacement, repair and maintenance costs [30,31]. Building structural integrity and occupant safety may also be impacted by excess moisture causing wood rot and corrosion or movement of metal fasteners, which can lead to building movement or structural failure [32,33].

It is widely acknowledged that dampness and mould in housing negatively impact the health of the occupants [34]. Numerous studies report adverse health effects in relation to dampness and/or visual mould within buildings, with a large proportion of this research focused upon asthma, allergic response, respiratory infections, respiratory symptoms and rare clinical conditions [1,25,35,36,37].

Very little attention has been given to unhealthy housing within an Australian context until recently [21]. It appears that Australian homes have previously been perceived as less problematic for dampness, cold or poor indoor air quality due to relatively young housing stock, generally good condition of that stock and a mild climate compared to other countries [21,38]. In addition, excess condensation, high humidity and mould appear to be accepted as a normal part of the residential home, especially in relation to bathrooms and windows [39,40], and occupants’ awareness of the potential health impacts from indoor mould is limited [39,40,41].

Despite international evidence showing that mould in housing likely relates to the synergistic effects of interrelated factors, recent Australian studies in this area appear to have been conducted in discrete disciplines while indicating emerging concerns:

- Architecture, building sciences and the construction industry exploring case studies and anecdotal concerns for excess condensation and mould in energy efficient housing [39,42];
- Building physics hygrothermal risk analysis confirming concerns for the risk of indoor mould in new housing typologies [43,44,45];
- Social sciences, public health and housing advocacy groups reporting susceptible populations at risk of poor housing conditions (cold, damp and mould), housing inequality, health impacts and socioeconomic disparity [46,47,48];
- A recent government inquiry into biotoxin-related illness reported impacts on health and economic well-being of occupants living in mould-affected housing [41].

To compound matters, as with many other countries, COVID-19 restrictions implemented in Australia since March 2020 have resulted in more Australians spending more time at home due to government-mandated lockdowns, work-from-home requirements, home-schooling and childcare, and an increase in preferences to continue working from home after restrictions ease [49,50].

As a critical starting point for addressing housing, mould and health synergies, this paper aims to develop a robust integrated review methodology for this topic and bring together the current state of evidence on the prevalence, risk factors and impacts related to reported mould-affected housing conditions in an Australia context. This process helps to identify key issues and gaps in the knowledge base and to highlight future research directions in order to inform Australian building, housing and health research, practice and policy. Without a clear understanding of the current state of evidence, there is little basis for informing evidence-based policy, increasing public awareness and mitigating the occurrence of mould in Australian housing. Both the process applied in this review and the findings are of relevance to the broader international community.

## 2. Materials and Methods

### 2.1. Literature Review Methodology 

An integrative review method was selected for this study, as it allows for a narrative synthesis of qualitative and quantitative studies for the purpose of presenting the current state of evidence on a particular phenomenon, contributing to theory development and to help inform practice and policy. This review method allows for drawing conclusions from a range of study methodologies and sources that bridge disciplines, while ensuring rigour, quality control and replication [51,52,53]. This study adopted the integrated review framework outlined by Toronto and Remington [53].

This integrative review seeks to answer the following research question:

*What is the current state of evidence on the prevalence, risk factors and impacts related to mould-affected housing in Australia?*

Sources of the literature were restricted to the English language and studies conducted within Australia. A multiple-disciplinary literature search strategy was adopted to capture the broad nature of the research inquiry. Publications were first sourced through a search of four broad-coverage academic electronic databases (Embase, PubMed, Scopus, ScienceDirect), with no date restrictions, conducted in February 2020 and again in September 2021. Due to the relatively small number of papers resulting from these searches, a grey literature search was then conducted across Australian Government agencies, data archives, and policy and advocacy websites for relevant reports. A list of the grey literature website sources is shown in Table 1. In addition, a manual search for additional studies was conducted using citation related reference screening from the identified academic papers, and a manual search of the research team’s database.

Search terms were initially organised into four themes: Place, Mould, Housing and Risk/Impact. The initial selection of search terms was identified following the screening of a major review paper on indoor dampness and mould by the World Health Organization (WHO) [1]. Additional search terms were added following the screening of keywords used within relevant articles within the research team’s database. The final search terms, with synonyms and syntax, are provided in Table 2. The search strings with Boolean operators used for each database are provided in the Supplementary Materials (Table S1).

### 2.2. Inclusion/Exclusion Criteria and Quality Appraisal

In general, publications were considered for inclusion if they had full-text availability, were related to original research and were published in English in peer-reviewed academic journals or conference proceedings, or by reputable organisations. Studies needed to report on indoor mould/mildew/fungi in relation to Australian dwellings (detached and semi-detached houses, townhouses, units/apartments) and include prevalence, risk factors or impact. Studies on both children and adults were included. A full breakdown of the study inclusion and exclusion criteria is provided in Table 3. Both positive and negative outcomes related to mould/mildew/fungi have been included.

While the quality appraisal of studies included within an integrative review allows for more rigour within the study review process, there is limited consensus on the best way to approach the appraisal of multiple study types and sources of data within public health [54]. Nor is there a consensus on whether to include all studies with varying degrees of assessed quality or to remove poor-quality studies and potentially bias the results or consensus on which tool to use for various types of study designs and grey literature [53].

Peer-reviewed academic literature and grey literature were assessed using the Mixed Methods Assessment Tool (MMAT) [55], while the Authority, Accuracy, Coverage, Objectivity, Date, Significance Checklist (AACODS) was used to evaluate the grey literature [56]. Each study was attributed an overall quality rating using an asterisk (*) when answering “Yes” to each question. The MMAT tool has a total of five questions, which equates to five asterisks (*****) if all questions answered “Yes”. A similar approach was used for the AACODS using a six broad theme question checklist. The lead author (L.C.) rated the domain criteria as “Yes”, “No”, “Unknown”, or “Not applicable” with the second author (W.M.) randomly checking 25% of the ratings to ensure consistency.

Due to the nature of this review in seeking to understand the current state of evidence, all studies that met the inclusion criteria were included regardless of each study’s quality. The studies’ quality appraisal results are included in Table 4 (summary of included studies) and a breakdown of the results is provided in the Supplementary Materials (Tables S2–S6).

### 2.3. Data Management and Extraction

All the studies identified were initially charted into a Microsoft Excel spreadsheet with basic details, author(s), year of publication, title and database, prior to screening. Studies identified for full-text screening were saved as PDFs to an electronic file and read electronically. All included studies were saved electronically to Mendeley Reference Manager and the articles’ key findings were extracted to word summary tables.

**Figure 1:**
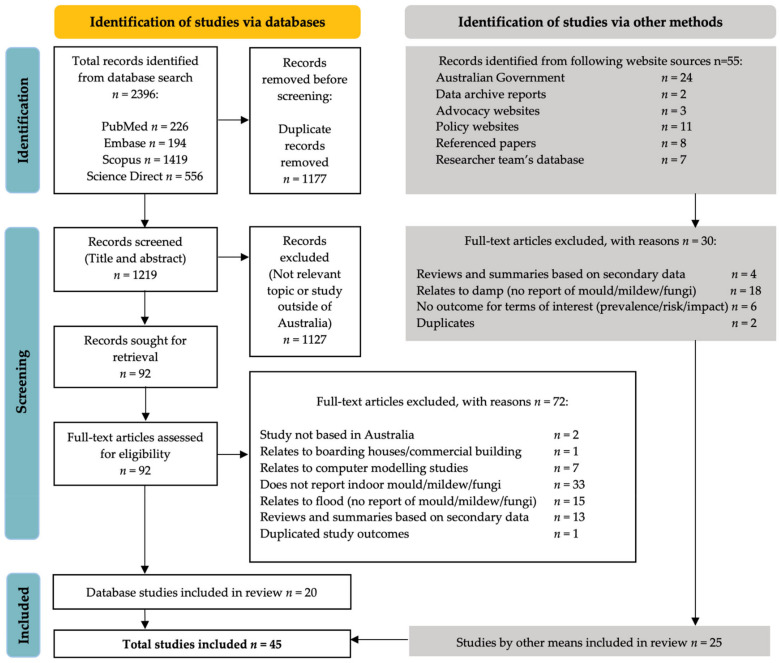
Preferred Reporting Items for Systematic Reviews and Meta-Analysis (PRISMA) flowchart of search results.

This search process was guided by the Preferred Reporting Items for Systematic Reviews and Meta-Analysis (PRISMA) flow diagram illustrated in Figure 1. A total of 2396 articles were identified from four electronic database searches (Scopus *n* = 1419, ScienceDirect *n* = 556, PubMed *n* = 226 and Embase *n* = 226). Following the removal of 1177 duplicates from the database search, 1219 records were screened by Title and Abstract, resulting in 92 articles remaining for full-text review. A further 55 grey literature studies identified via other methods were added to the full-text review. Forty-five studies ultimately met the inclusion/exclusion criteria following the full-text review. A summary list of all the included studies is provided in the Supplementary Materials (Table S7).

## 3. Results

### 3.1. Profile of Included Literature

The final 45 studies selected for this review varied significantly in their study design and included 11 cohort studies (24.4%), 9 cross-sectional studies (20%), 8 prevalence studies (17.8%), 3 qualitative descriptive studies (6.7%), 3 mixed methods building industry research reviews (6.7%), 7 case studies (2 human (4.4%) and 5 housing (11.1%)), 2 mixed methods studies (4.4%), 1 interventional housing study (2.2%) and 1 governmental inquiry research summary report (2.2%). Twenty-nine studies (64.4%) were published in an academic journal or publication and 16 studies (35.6%) were obtained from other sources indicated in Table 1. A breakdown of the study characteristics, study design types, study topics, geographical distribution and quality appraisal is indicated in Table 4.

### 3.2. Study Topics Included in the Literature 

Five main study topics, based on study titles, emerged from the included literature, as shown by rank order in Table 5. It clearly shows that building characteristics is the most frequently studied topic, and occupant behaviour is the least studied topic. Most health-related topics reported specific health conditions: asthma, allergy, respiratory conditions, hypersensitivity pneumonitis, domestic allergic alveolitis, and only five studies reported multiple-symptom health effects and/or environmental conditions. Nine study topics investigated housing conditions and socioeconomic housing factors and their relationship to gastrointestinal infections, perceived health, tenure or renting. The most recent studies, focusing on housing and COVID-19, illustrate the lived experience, housing, and health impacts and challenges.

### 3.3. Chronological Distribution of Included Literature 

**Figure 2:**
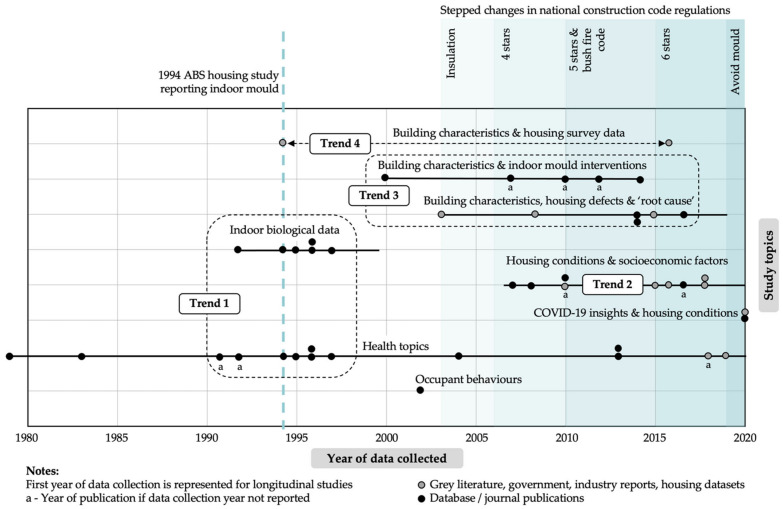
Chronological distribution of reviewed studies by theme and data collection year.

The chronological distribution of all included studies is illustrated in Figure 2. First it shows the date that data collection commenced or the publication date of each publication, organised into themes: building characteristics (housing survey data, indoor mould interventions, housing defects and “root cause”), indoor biological data, housing conditions and socioeconomic factors, COVID-19 insights and housing conditions, health topics, and occupant behaviours. Overlaid on this timeline is:

- The stepped changes in building energy efficiency regulations in Australia that have focused on improvements in the thermal efficiency of the building envelope (represented by the star rating [45,96]);
- The cessation of mould reporting by the Australian Bureau of Statistics.

This figure clearly illustrates significant changes and trends in “who” and “how” the subject of residential indoor mould has been researched over time. Four main trends are evident:

1. The investigation of indoor air quality in housing and biological data in relationship to health was mainly undertaken in the mid-1990s;
2. There are three times more studies exploring socioeconomic factors/circumstance relating to housing conditions than that of epidemiological, clinical and health studies since 2007;
3. As building regulations moved towards more energy efficient housing and bushfire housing safety requirements, building sciences, architecture, property, law and the building/mould industry start investigating interventions, defects and “root cause” of moisture-related building issues in built environment;
4. There is a 22-year gap in capturing or reporting indoor mould in population-based housing studies. This may indicate an incorrect assumption, until relatively recently, that enhancements in building regulations had “fixed the problem” of mould in houses (and hence the perceived lack of need to collect this data). Alternatively, it may be an indication of the consequences of the funding restrictions placed on the data collection agency (and hence the inability to collect all of the data about housing that was previously funded).

These trends highlight both a fragmented approach to studying indoor mould in Australia and considerable gaps in current health/housing biological data and housing survey data over the course of time. By contrast, recent study trends shift towards housing conditions and its relationship to socioeconomic factors and that of indoor mould interventions, defect and “root cause” studies. This shift towards housing defects and “root cause” studies, and housing conditions and socioeconomic factors is an interesting finding for both housing and building policy, and by simply implementing and reporting on regular housing condition surveys the extent of the problem of indoor mould can be better understood.

### 3.4. Geographical Location of Included Literature

**Figure 3:**
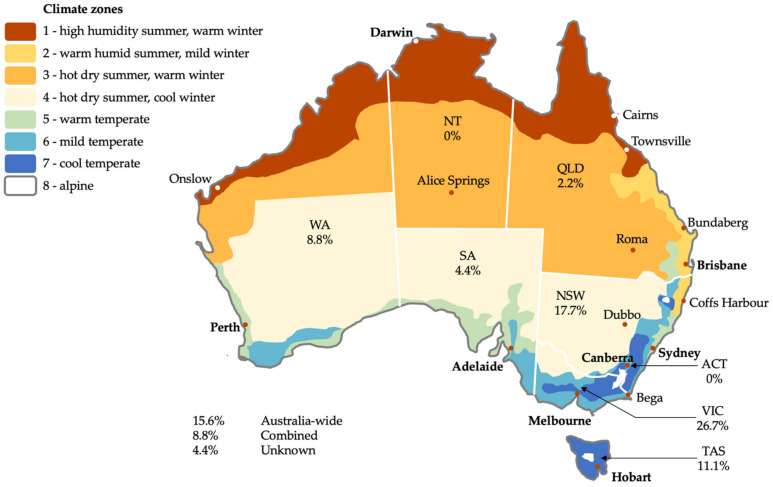
The geographical distribution of all included studies in relation to climate zones.

Australia is a federation of eight states and territories which intersect multiple climate zones. Eight broadly classified climate zones are illustrated in Figure 3, which have been classified in the National Construction Code (NCC) for the purpose of energy efficiency regulatory provisions [97]. Australia is a large continent (almost 7.7 million km^2^) that stretches 3860 km from north to south (latitudes 10°41′ S to 43°38′ S). This means that the climate varies greatly between climate zone 1 in the northern tropics (high humidity, warm summers) to that of the alpine conditions in climate Zone 8. While there is a National Construction Code, each state and territory has the legislative authority to adopt and enforce building regulations within their jurisdiction. The federal government has no jurisdiction over buildings. About 85% of the population lives within 50 km of the coastline [98].

Geographically, seven studies (15.6%) were across all states and territories. The vast majority of the studies were based in Victoria (VIC) (*n* = 12, 26.7%) and New South Wales (NSW) (*n* = 8, 17.7%), followed by Tasmania (TAS) (*n* = 5, 11.1%), Western Australia (WA) (*n* = 4, 8.8%), South Australia (SA) (*n* = 2, 4.4%) and Queensland (QLD) (*n* = 1, 2.2%). Four studies were based in multiple states (8.8%) while only two studies (4.4%) had an unknown location. Although there are seven Australia-wide studies, the majority of studies (*n* = 25, 55.5%) are located around the cooler/mild/warm temperate climates zones within TAS, VIC and NSW. Outside of the Australia-wide studies, South Australia and Queensland have limited study coverage, and the Northern Territory (NT) and the Australian Capital Territory (ACT) have no study coverage. This equates to over half (52.8%) of the landmass of Australia having none or very limited dedicated study coverage (NT, QLD, SA and ACT), while 3% of the landmass has the highest concentration of dedicated studies (26.7% in VIC). In addition, there is no study coverage in some of the most vulnerable climates for indoor mould being that of the tropical location of climate zone 1, and only one dedicated study in the sub-tropical location of climate zone 2.

Regional (coastal or inland) and remote areas, which often have different housing qualities and access to different construction types, housing materials and labour expertise compared to urban/city locations, have no dedicated study coverage. As a consequence of a vast geographical expanse, Australia has an incredibly diverse range of climate zones, housing typologies, construction typologies and practices, different building regulations and different ways of living in housing. Despite this, there are a surprisingly limited number of studies exploring these vast differences in Australian housing.

## 4. Discussion

The main factor for mitigating dampness and mould in the indoor environment is by limiting liquid moisture and moisture as vapour, within building materials [99], within a building system or in the building [100]. Understanding the many causes of excess moisture and preventing these risks from occurring in the first place is the key to mitigating the risk of exposure to mould, dampness and mould-related indoor contaminants. Previous international research described eight broad categories of risk for indoor mould and are used to unpack the coverage of literature, which includes: (1) climate; (2) housing conditions; (3) socioeconomic factors; (4) building characteristics; (5) occupant behaviours; (6) location; (7) building maintenance; and (8) urbanisation [1,23,24]. The first five of these are used in the following discussion relating to the selected literature. In this review, no Australian literature was found relating to three of these risk categories (climate, urbanisation and building maintenance), indicating a lack of coverage of critical, known risk categories relevant to contemporary housing development trends, the management of poor housing conditions and the weather.

As a general finding, the most common reported relationship (*n* = 35, 80%) was that of indoor mould/mildew/fungi and risk factors or associations for either housing conditions, socioeconomic circumstance or building characteristics. This was closely followed by the reported prevalence (*n* = 22, 48.9%) of visual mould/mildew or measured fungi in housing and impacts from reported indoor mould focused on the perceived or measured health impacts (*n* = 21, 46.7%). The following sections provide more insights into the included literature.

### 4.1. Climate and Risk of Indoor Mould 

The majority of all the included studies (*n* = 30, 66.7%) report indoor mould while investigating housing conditions (*n* = 12), or report indoor mould while investigating building characteristics (*n* = 18). The climatic distribution of included studies reporting housing conditions and building characteristics is illustrated in Table 6.

From the studies investigating housing conditions, the majority of studies (*n* = 7, 58.3%) did not report the study locations in detail, making it impossible to ascertain their climate locations. Of these seven studies, two studies were from all states and territories, two studies were based in SA, one study was based across NSW, VIC and SA and two studies were from unknown locations. The remaining five studies were located in climate zones 5 and 6—warm/mild temperate zones.

From the studies investigating building characteristics, the majority of the studies (*n* = 13, 72.2%) were located within either the warm and mild temperate climate zones (5 and 6) (*n* = 10) or the cool temperate climate zone 7 (*n* = 3). Four studies (22.2%) did not report the study locations in detail to infer their climate zone. Of these four studies, one study covered NSW, QLD, VIC, WA and unknown locations, one study covered VIC, TAS and NSW, one study covered NSW, QLD and VIC and one study included all states and territories. Only one of the national studies that included building characteristics included a breakdown of the results by climate zone [39]. Outside of this study, there are no climate and location-specific studies investigating relationships between housing conditions, building characteristics and risk of indoor mould for the warmer and humid climate zones (climate zones 1 to 4) and alpine regions (climate zone 8).

**Figure 4:**
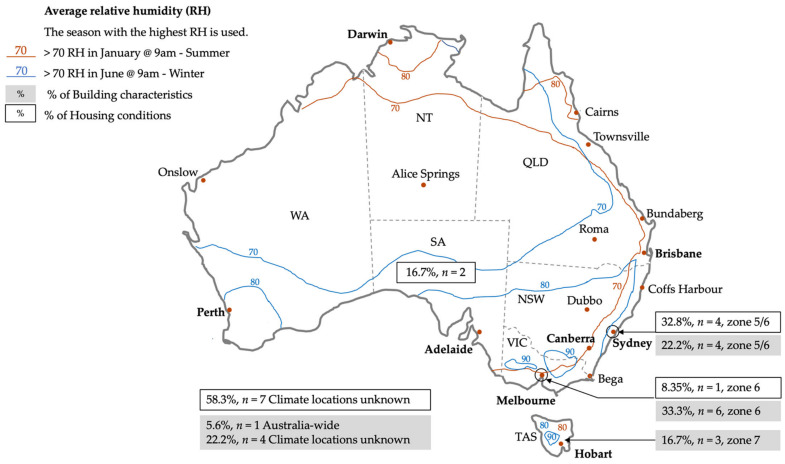
The distribution of housing conditions and building characteristic studies in relation to average RH.

Figure 4 illustrates approximate Australian seasonal average relative humidity (RH) values and the distribution of studies by climate zone. In summer, high humidity levels (>70% RH) are not only located in the warmer regions of northern NSW, QLD, NT and northern WA (climate zones 1 and 2) but also extend along the eastern coastal communities in summer months as far as VIC and TAS (climate zones 6 and 7). In the cooler winter months, high humidity levels (>70% RH) extend from southern WA, southern SA, VIC, ACT, NSW and TAS, through to north QLD [101].

Surprisingly, only three small studies based in climate zone 5/6 focused on measuring indoor conditions, RH and the risk of indoor mould, illustrated in Table 7. Data gathered from housing in warm/mild temperate locations appear to be susceptible to a range of RH levels ranging from RH > 60% to RH > 80% in bedrooms and the risk of indoor mould. High wall surface temperatures compared to the room air dew point temperature (for example, when a hot temperature is lowered quickly with air-conditioning) was also reported to be a risk for indoor mould, while mould growth modelling tools based on RH levels failed to predict the risk of mould in a small number of case study houses [73].

It is interesting to note that potential indoor mould is both dependent on, and influenced by, indoor and outdoor climate conditions such as temperature, available moisture and humidity [1,102]. Australian housing is highly susceptible to outdoor RH levels due to a number of reasons:

- Houses have subfloor ventilation and naturally ventilated roof spaces;
- The opening of windows to cool a home is common on warmer days;
- Residential buildings are not fully sealed or fully mechanically cooled or ventilated.

Despite this, there are surprisingly very limited housing studies exploring real-time and longitudinal housing climate conditions, temperature, available moisture and humidity and their relationship with housing conditions, location and/or building characteristics and the occurrence of indoor mould. From the handful of studies that do capture real-time data, they are only based in the cooler climates. One study in Sydney NSW (climate zone 5/6) specifically measures the risk of mould in five freestanding houses [73], two studies in Tasmania (climate zone 7) investigate excess condensation, mould and moisture damage to five freestanding houses [42,82] and only one case control study in Victoria (climate zone 6) explores homes (*n* = 30) for energy costs, perceived health and temperature outcomes in winter [70]. A national representation of real-time and longitudinal housing conditions for indoor temperature, available moisture and humidity for all building typologies (new and older housing) is desperately needed to understand their relationship with the occurrence of indoor mould.

### 4.2. Housing Conditions and Risk of Indoor Mould

Housing conditions have been mainly measured in Australian housing survey studies by using a question similar to the Australian Housing Conditions Dataset, ADA questionnaire [103]. The housing survey asks the question: “Does this dwelling have any MAJOR building problems?” and provides the following drop-down options: rising damp, mould, cracks, sinking/moving foundations, sagging floors, walls/windows out of plumb, wood rot/termite damage, electrical problems, roof defects, others please specify and no problems. The advantage of using this question is that it allows for longitudinal analysis and comparisons between studies over time. However, surprisingly, 16 reports located on the Australian Bureau of Statistics (ABS) and Australian Institute of Health Welfare (AIHW) websites that were identified for screening and described the use of this housing condition question or similar, did not report indoor mould as a variable in their reports. They were therefore excluded from this review.

The first time this question appears is in the first building characteristics, cost and condition survey capturing the data from 1994 and published in 1996, where indoor mould and its relationship with building characteristics is reported in detail [60]. This question then continues to be used in a variety of housing and social trends surveys by ABS every two to four years, without reporting the mould variable. Since 2012, the AIHW have also included this question in their social housing surveys, but without reporting the “mould” variable. It is not clear why indoor mould is not reported in these housing condition studies. Feasible explanations include:

- The question does not allow for the variable “mould” in the answer?
- The variable is not statistically significant enough to be reported upon.

The studies that did use this question (or similar) and did report mould are included in the following discussion around housing conditions. It discusses the interactions between the conditions of housing, poor housing conditions and housing tenure, socioeconomic status and exposure (longer hours of occupancy due to COVID-19). Across all the included studies, the most commonly described poor housing conditions included: visible mould or mildew on walls, ceilings and windows; absence of functional heating or cooling; rising damp; peeling paint; leaking roofs; leaking pipework and ceilings; broken amenities; structural problems; major cracks and overcrowding.

#### 4.2.1. Housing Conditions, Rental Housing and Risk of Mould

Poor housing conditions from mould, dampness, cold and cracks were reported in twelve (26.7%) of the included studies with rental properties being the most represented. Baker and Daniels [46] reported that up to 78% of rental properties (*n* = 14,486) across a mixed tenure of private rental, public and community housing were reported as “very poor” quality housing by occupants, indicating the widespread problem of poor-quality housing in the rental market. Cracks in walls and floors (40%) was the most commonly reported problem by households, followed by indoor mould (27%), dampness (21%), and not being able to keep warm in their home during cold weather (23%).

The prevalence of poor housing conditions in rental households and the reporting of problems with indoor mould were supported by three further studies. One study by the advocacy group Choice et al. [47] reported that 51% (*n* = 1547) of renters were living in a home that is in need of repairs and 35% of renters had experienced problems with mould in their bathrooms and 20% in their bedrooms. Poor-quality housing conditions continued to play a part in private rental homes that experienced leaks or flooding (21%, *n* = 1005) [58], with 20% of households reporting mould that is difficult to remove or reappears and 18% reporting difficulty in keeping the property warm or cool. Interestingly, a secret shopper survey by the Victorian Council of Social Services (VCOSS) [61] found that 12% of private rental homes inspected (*n* = 116) were uninhabitable, 10% had no heating, 19% reported visible and extensive mould (majority in the bathroom), and lack of ventilation was a concern (53% of homes had one room with no ventilation and 29% had two rooms where windows were unable to be opened).

These findings suggest that rental properties are at a significant risk for potential problems with both poor housing conditions and indoor mould. This is a public health concern given that approximately one in three Australians rent [46] and Australian rental properties make up 32% (*n* = 2.6 million households) of Australia’s housing stock. Of this 32%, 26% (*n* = 2.1 million households) rent from private landlords and 3.7% (300,000 households) rent from public social housing authorities [104].

#### 4.2.2. Housing Conditions, COVID-19 Insights and Risk of Mould

Increased time at home due to COVID-19 factors were reported to have both protective and negative consequences with regard to mould, dampness and building cracks [57]. On the one hand, the stay-at-home orders made it more difficult to overlook housing physical damage and defects and promoted the need for repair. On the other hand, others endured prolonged exposure to dampness or mould, delayed repairs, or experienced delays in landlords’ ability to address dampness and mould issues. The reasons for this delay in repairs or why repairs were previously overlooked is unknown, but may be a downstream effect of COVID-19 lockdowns, lack of landlord funds, delays in insurance claims, limited access to properties, shortage of trades or delays in reporting a problem due to fear of eviction [46].

### 4.3. Housing Conditions, Socioeconomic Circumstance and Risk of Mould

Of the 12 studies that investigated housing conditions for indoor mould, damp and cold housing, eight studies discussed the perceived health impacts and seven studies reported a disproportional prevalence of poor-quality housing and indoor mould for those who are most socioeconomically disadvantaged.

Households living in unaffordable rental situations reported mould in 39% of homes [46], with the prevalence of reported mould being significantly higher in social housing (42% to 50%) [46,48,78] compared to the private rental market (19% to 33%) [48,58,61,78]. Interestingly, owner/mortgagee households reported mould in only 13% of homes [48].

Households with children were also disproportionately at risk from poor-quality rental housing and indoor mould. Among households with children who rent, 39% of couples with children and 44% of single parent families reported living with three or more housing condition problems. In total, 50% of families with children in social housing reported multiple housing problems including mould and dampness [46,47].

A similar story unfolds for households from Indigenous or refugee and asylum seeker backgrounds where cold, dampness, mould and overcrowding were commonly used to describe conditions and factors that had the greatest negative effect on occupant health [87,88]. A high prevalence of housing problems was reported among urban housing for Indigenous Australians [76] with social housing for Indigenous households accounting for 43% (*n* = 600) of the damp and mildew-affected homes [48].

It is widely recognised that poor housing quality and poorly maintained housing are risk factors for dampness and mould and damp housing conditions increase the risk of asthma related to allergens, mould and house dust mites [1,105,106]. These findings strongly suggest that those in poor socioeconomic circumstance in Australia are more at risk of negative health impacts associated with poor-quality housing conditions, including indoor mould.

The prevalence of asthma in Australia is surprisingly one of the highest in the world [107] with 1 in 9 Australians (11%) having asthma [108]. The data shows that asthma is highest in those living in the lowest socioeconomic areas (13%) compared to those living in the highest socioeconomic areas (10%), and that the asthma prevalence in indigenous populations is 1.6 times that of non-indigenous Australians [109]. While it is acknowledged that not all asthma is causally linked to mould, there does seem to be strong evidence supporting the need to address the problem of poor housing conditions in general and reduce indoor mould and dampness. Such steps would be a positive move towards reducing Australia’s asthma rates, reducing the cost of asthma on the health system ($770 million in 2015–2016) [108] and creating healthier housing for those who are already socioeconomically disadvantaged.

### 4.4. Building Characteristics and Risk of Mould

Building characteristics and risk factors for indoor mould in the Australian context are illustrated in Table 8, by type of risk and if the data was collected before or after 2003 with the introduction of housing energy efficiency provisions.

Two different pictures emerge within the included studies for building characteristics and risk of indoor mould. Older homes with data collected prior to 2003 include age-related conditions such as: age of home greater than 40 years, poor housing conditions, cracks in cladding, water-damaged floorboards, roof leaks, water intrusion and carpets older than 5 years. Older housing typologies such as housing with stumps, double brick or brick veneer walls, inadequate ventilation or air movement, limited insulation, lack of kitchen or bathroom exhausts and cold rooms were also at risk from indoor mould.

By contrast, the majority of the studies capturing housing data after 2003 report a shift in risk to that of the use of air-conditioning, building leakage (internal and external) attributed to construction defects and concerns for surface/interstitial condensation in code-compliant houses, apartments and units.

The risk of mould in split-system air-conditioning units occurs due to a lack of maintenance, while the risk of mould in a room or wall cavity occurs due to temperature differences between a conditioned room and an unconditioned room or space. Latent conditions of hidden mould reside in areas related to building defects, such as failed waterproofing or problems with gutters that affect the wall, roof or floor cavities but are not visible for detection until the problem has been occurring for a long period of time [39,92]. In addition, one study reported a national concern for up to 40% of homes built since 2003/4 being at risk of excess condensation resulting in indoor or interstitial mould [39]. Case studies of housing in cooler climate zones investigating interstitial mould within the building envelope [42,82] and building physics hygrothermal risk analysis studies (excluded from this review) both currently support this concern [43,44,45,82,102,110,111,112].

Understanding the national prevalence of contemporary building characteristic risks and the “root cause” associated with indoor mould is not conclusive from the limited geographical and climatical coverage of the included studies.

### 4.5. Occupant Behaviours and Risk of Mould

Considering the critical role an occupant plays in managing ventilation, thermal comfort, condensation, cleaning, household maintenance and the risk of indoor mould, this topic is surprisingly understudied in Australia. The majority of the included studies (75%) discussing the relationship between occupant behaviours and the risk of indoor mould are based on older housing typology (20 years old or more) and were collected before the introduction of newer housing built to energy efficiency standards. However, the role the occupant plays in managing indoor mould, housing ventilation and cleanliness was the most reported relationship that both increases and reduces the risk of mould in housing, illustrated in Table 9. On the one hand, highly naturally ventilated homes and highly cleaned homes decreased the risk of indoor mould [40,67] while, on the other hand, the opening of windows increased indoor fungal levels [67]. One study examining the relationships between indoor mould, health effects and occupant behaviours reported that highly naturally ventilated and highly cleaned homes decreased the presence of moulds in the homes, but surprisingly highly cleaned homes increased the prevalence of wheezing and rhino-conjunctivitis in children [83].

Past experiences from older occupants having grown up sleeping on enclosed verandas or “sleep-outs” influenced current protective behaviour measures to keep windows or doors permanently open to avoid the risk of mould and improve breathing in newer homes [70]. Lack of occupant awareness on the cause of indoor mould was also reported among 60% of households who removed mould growth on a regular basis but only 23% of these occupants considered their home to have moisture-related issues [40].

Recent studies report a reluctance to open windows due to energy costs and a lack of occupant awareness of their behaviours and role in reducing condensation [39]. More research is needed to confirm if this shift in behaviour is due to how occupants manage newer housing typologies compared to older housing or simply due to lack of study coverage over time. Considering one person generates eight litres of moisture per day from daily household activities [45] and newer housing typologies have been increasingly changing since 2003, there are surprisingly limited empirical data investigating occupant behaviours and risk of indoor mould.

### 4.6. Prevalence of Indoor Mould Conditions in Australian Housing

**Figure 5:**
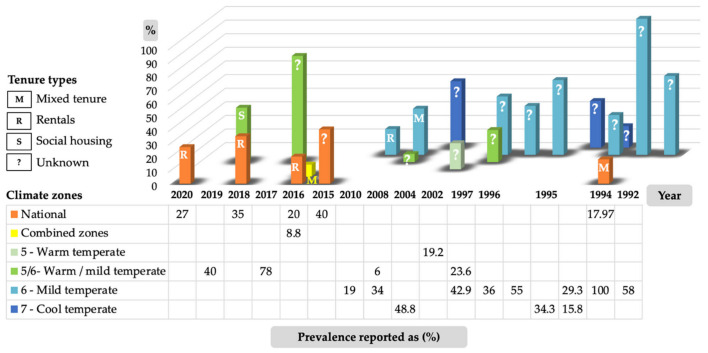
Prevalence of reported indoor mould/mildew/fungi conditions within the included studies by climate zone and data year.

The prevalence of mould/mildew/fungi-affected dwellings was reported in approximately half of the included studies (*n* = 22) (48.9%) and is illustrated in Figure 5 by climate zone and the year the data were collected. However, since 1994, the national prevalence and geographic distribution of mould in Australian homes is still essentially unknown.

The only national cross-sectional, population-based housing survey that reported indoor mould conditions for all housing tenures was conducted in 1994. This study by the ABS [60] reported that 17.97% of homes (*n* = 14,456) had mould or mildew. A more recent study by Baker et al. [91] capturing data in 2016 from 4501 households in NSW, VIC and SA reports that the prevalence of indoor mould for these locations has improved for mixed tenure situations. Surprisingly, over half (51.9%) of these dwellings experienced cracks in walls/floors, 13.7% of dwellings reported roof defects and 13.9% reported rising damp, however only 8.8% of dwellings reported problems with mould. The reason for this lower prevalence of indoor mould compared to that of the majority of studies in Figure 5 may be related to tenure type, with 87.9% dwellings being owner occupied compared to 10.2% rental dwellings. However, without further insights and comparable data collected across all studies, this is hard to confirm and highlights the need to improve the way we collect data.

Reporting comparable data in future academic and grey literature is strongly recommended for: the year(s) when the housing data is collected, tenure, housing type, age of the property, housing typology and other critical information, for example, climate zone, location, building characteristics, and if the property had undertaken renovations, repairs or maintenance, especially for moisture-related problems. This would conceivably provide more meaningful insights for policy decisions when investigating the prevalence, root cause and risk of indoor mould in relation to the building envelope design, housing typology and housing and building regulatory provisions. Longitudinal studies (for example, surveying the same dwellings every 5 years or so over a 30–40 year period) are recommended to help identify the impact of home renovation and maintenance activities and monitor housing conditions.

Although the current evidence is inconclusive to establish a full geographical, climatical and typological representation and comparable study methods, the results suggest that the prevalence of mould/mildew/fungi continues to be an unresolved problem in Australia housing.

### 4.7. Health Impacts Related to Reported Mould/Mildew/Fungi

Internationally, there has been considerable research undertaken in the area of respiratory health, allergies and respiratory infections following exposure to dampness and mould [1]. Similar health impacts following exposure to mould/mildew/fungal indicators have also been explored within Australia, with the majority of included health studies (*n* = 12, 26.7%) supporting a causal relationship with mould/mildew/fungal indicators and asthma, wheeze, cough, respiratory and clinical associations of domestic allergic alveolitis and hypersensitivity pneumonitis. Reported relationships between mould/mildew/fungal indicators and health effects are illustrated in Table 10.

Seven of the included studies (15.6%) reported allergy, atopy, gastrointestinal, mood, depression and pain in relation to indoor mould/mildew/fungal indicators. While allergies in children [40] and an increase in allergy to fungi [62], pollen and dust mites [90] were reported to be related to indoor mould indicators, houses with current indoor levels of fungi were protective and reported lower risk of allergy to fungi [64]. Interestingly, highly cleaned homes reported a higher prevalence of current wheezing and rhino-conjunctivitis [83].

The remaining five studies (11.1%) reported a mixed variety of symptoms similar to that of Sick Building Syndrome (SBS) and overlapping environmental illness conditions in relation to indoor mould. The earliest report in Australia of SBS-type symptoms in relation to indoor mould was reported by Robertson [65] in the early nineties, reported as hypersensitivity in both the occupants and the investigators. Sick building syndrome has been described as a collection of varying non-specific symptoms that are temporal in nature and result in respiratory symptoms, skin/dermal conditions, flu-like symptoms, eye conditions, asthma, headaches, nausea, tiredness and malaise [116]. Avoidance of the problematic indoor environment or relief from the environment, for example at the weekend, is reported to resolve the symptoms of SBS [117]. More recently, international research suggests that reversible SBS has the ability to proceed to irreversible hypersensitivity to dampness and/or mouldy indoor conditions that affect many body systems. This is known to present as multiple symptoms and be linked to autoimmunity and such conditions like postural tachycardia syndrome (POTS) and myalgic encephalomyelitis/chronic fatigue syndrome (ME/CFS) [118,119]. The comorbidity of a biotoxin or mould-related illness is also reported to intersect with multiple chemical sensitivity (MCS) [94], tick-borne illness [94] and ME/CFS [59,94] in recent Australian studies.

Emerging anecdotal evidence from the included studies reports that perceived health effects related to living in housing with dampness or visible mould extend beyond previously well-studied health associations to that of changes in mood, sadness, depression [57,87], pain [87,90] and other cognitive and physical symptoms for susceptible individuals [41,65,90].

Individuals responding to the Australian Government’s inquiry into biotoxin-related illness [41] reported multiple-symptom characteristics, in relation to indoor mould, as debilitating and chronic in nature, and resulted in severe impacts on both economic well-being and finding safe housing [41]. Further research is critically needed to understand these perceived multiple-symptom health effects and their relationship to biological components that are present in mould-affected housing.

### 4.8. Gaps and Implications for Research, Practice and Policy

The purpose of this review was to bring together the full range of research on the prevalence, risk factors and impacts related to mould-affected housing in an Australian context in order to disseminate the findings and identify research gaps and implications for building, housing and health research, practice and policy.

This review highlights a general lack of geographical and climatical coverage, especially for warmer tropical/sub-tropical areas and regional locations. This is compounded by the sheer size of the country, a lack of national housing survey data and lack of consistency in the type of data collected and by a fragmented approach to how mould-affected housing has been studied over time. Notwithstanding these coverage limitations, synthesis of the evidence reveals that indoor mould in Australian housing exists in a complex interrelated and interdependent system of risk factors between the built environment (poor housing conditions, building defects, lack of ventilation and surface/interstitial condensation), occupant behaviours (lack of awareness, limited ventilation and impact of energy costs), socioeconomic factors (unaffordable rental situations, social housing and rental housing, occupants from Indigenous or refugee and asylum seeker backgrounds), household structure (families with children in social housing and single parent families who are renting) and implications of regulatory provision and protection policy (housing energy efficiency provisions or public health directions).

These findings support previous observations in energy-related risk factors for indoor mould [120] and recognition of the complexity of interrelated factors, for example, the buildings’ ability to be heated or ventilated effectively (building design), and energy affordability (socioeconomic factors), along with the occupants’ reluctance in opening windows to increase natural ventilation (occupant behaviours), which impacts the risk of indoor mould in colder climates. Although Australian housing and construction typologies are different from that of other countries, similar comparisons for the risk of indoor mould related to excess surface condensation can occur in some energy efficient houses [121]. This may be due to a combination of lower air change rates, a rise in RH and excess surface condensation [122]. In addition, it is well understood that lower-income households [123], displaced populations, Indigenous people and single parent families have a greater risk of being exposed to poor housing conditions, overcrowding, dampness and indoor mould [124]. This review highlights the need for a holistic research approach, with quantitative and qualitative methods, to understand and address these complex relationships.

Future research would benefit from adding two additional broad-risk categories for indoor mould that have been overlooked or observed in Australia. Firstly, for countries with significant travel distances or housing located in regional or remote locations, extending the eighth category of risk for “Urbanisation” to include “Regionalisation”. Secondly, introducing a ninth risk category called “Policy” for nations who have experienced a national increase in the time spent at home or are in the process of developing energy efficient housing regulations or making significant changes to building typologies.

Further to the discussion, we highlight a need for future work to address the following research and practice needs:

- Implement national and climatical coverage of real-time data (temperature, available moisture, humidity) and “root cause” case study investigations for indoor mould in both older housing and code-compliant homes.
- Investigate the relationship between occupant behaviours, housing maintenance and the occurrence of indoor mould in poor-quality housing.
- Establish a national longitudinal housing condition survey that includes indoor mould, renovations and maintenance.
- To continue to use the question “Does this dwelling have any MAJOR building problems?” or similar in current longitudinal housing studies and report on the “mould” variable.
- Investigate the impact and characteristics of multiple-symptom health effects and their relationship to the biological components that are present in damp housing conditions.

With uncertainty around the cause, extent and prevalence of indoor mould in code-compliant housing, this study suggests that building regulatory authorities adopt the precautionary principle before moving forward with any future changes to housing energy efficiency provisions that have the potential to increase the risk of surface/interstitial condensation or indoor mould.

*“The precautionary principle, proposed as a new guideline in environmental decision making, has four central components: taking preventive action in the face of uncertainty; shifting the burden of proof to the proponents of an activity; exploring a wide range of alternatives to possibly harmful actions; and increasing public participation in decision making.”*

[125]

## 5. Conclusions

This is the first study to use an integrative review methodology to collate and unpack the current state of evidence on the prevalence, risk and impact from indoor mould in Australian housing. This comprehensive holistic review process can be replicated in other countries.

This study reports that the perceived emerging health effects following exposure to dampness and mould extends beyond accepted health associations such as respiratory symptoms, asthma and infections to that of a chronic multiple-symptom presentation. Our scientific understanding of these multiple-symptom health effects is limited globally and, within an Australian context, is unknown. Evidence on the extent, the prevalence of indoor mould and the relationship between building characteristics and the risk of mould is not conclusive on a national level for all residential dwelling types. This is because of a general lack of geographical and climatical coverage.

Like other countries, Australia has experienced significant changes over the last 20 years in housing and building policy regulations, the adoption of energy efficient housing provisions, a shift towards building apartments and an increase in single dwelling housing production in addition to a change in how occupants use their home. So as to provide healthier housing, future research into mould-affected housing is critically important to support regulatory decisions, especially since Australia experiences climate vulnerability, extreme weather events and sees a shift towards a greater number of Australians spending more time at home.

## Figures and Tables

**Table 1:** Source types and location of grey literature.

Type of Grey Literature	Agency/Group	Website Link
Australian Government	Australian Bureau of Statistics (ABS)	www.abs.gov.au/ (accessed on 1 November 2021).
	Australian Institute of Health Welfare (AIHW)	www.aihw.gov.au/ (accessed on 18 November 2021).
	Australian Building Codes Board (ABCB)	www.abcb.gov.au (accessed on 5 November 2021).
Data Archive	Australian Data Archive (ADA)	www.dataverse.ada.edu.au/ (accessed on 4 November 2021).
Advocacy websites	Housing advocacy group	www.shelter.org.au/ (accessed on 19 November 2021).
	Environmental sensitivities advocacy group	www.anres.org/ (accessed on 5 November 2021).
Policy websites	Australian Housing and Urban Research Institute (AHURI)	www.ahuri.edu.au/ (accessed on 8 November 2021).
	Analysis, Policy & Observatory	www.apo.org.au/ (accessed on 1 November 2021).

**Table 2:** Search terms by theme used for literature search.

Place	Mould	Housing	Risk/Impact
Australia	Mould	Hous *, House, Housing	Health
	Fungi	Indoor	Well-being, Well-being
	Mildew	Home *	Cost *
	Condensation	Dwelling *	Impact *
	Flood *	Residen *	Hardship *
	“Water damage”	Residential	Economic *
	“Water damage”	Residence	Financial
	Damp *	Building *	Risk *
			Perception *

* Used to capture permutations.

**Table 3:** Study inclusion and exclusion criteria.

Inclusion Criteria	Exclusion Criteria
Australian residential dwelling (detached and semi-detached single family homes, townhouses, units/apartments)	Houses or studies not based in Australia. Boarding houses or commercial buildings
Occupants (all ages), expert opinions or housing data (including biological)	Building computer modelling studies
Reports indoor mould/mildew/fungi	Does not report indoor mould/mildew/fungi
Data/information quantitative, qualitative, case studies relating to prevalence, risk factors or impact and reported indoor mould/mildew/fungi	No data or unclear outcome relating to prevalence, risk factors or impact for reported indoor mould/mildew/fungi
Any study design providing original data on housing conditions/occupants experiences or housing condition data from defects or insurance claims that are publicly available	Reviews and summaries based on secondary data
Full text available	Full text not available

**Table 4:** Summary of study characteristics *n* = 45.

Study Details	Study Categories		*n* (%)	Ref
Geographical distribution	Australia-wide		7 (15.6%)	[39,46,47,57,58,59,60]
	Victoria (VIC)		12 (26.7%)	[40,61,62,63,64,65,66,67,68,69,70,71]
	New South Wales (NSW)		8 (17.7%)	[48,72,73,74,75,76,77,78]
	Tasmania (TAS)		5 (11.1%)	[42,79,80,81,82]
	Western Australia (WA)		4 (8.8%)	[83,84,85,86]
	South Australia (SA)		2 (4.4%)	[87,88]
	Queensland (QLD)		1 (2.2%)	[89]
	Australian Capital Territory (ACT)			
	Northern Territory (NT)			
	Combinations: (VIC, TAS, NSW, QLD, SA, WA)		4 (8.8%)	[41,90,91,92]
	Unknown		2 (4.4%)	[93,94]
Quantitative Non-randomised Studies 18 (40%)	Cohort Studies		8 (17.8%)	[48,62,63,64,77,79,80,89]
	Cross-Sectional Studies		7 (15.6%)	[66,67,68,69,74,81,83]
	Case Control (houses)		2 (4.4%)	[85,86]
	Intervention Study (houses)		1 (2.2%)	[84]
Quantitative Descriptive Studies 18 (40%)	Prevalence Studies		8 (17.8%)	[46,47,58,61,65,71,90,94]
	Case Series (houses)		3 (6.7%)	[42,73,82]
	Cohort Studies		3 (6.7%)	[40,59,78]
	Cross-Sectional		2 (4.4%)	[91,95]
	Case Report (human)		1 (2.2%)	[93]
	Case Control (human)		1 (2.2%)	[75]
Mixed Methods Studies 5 (11.1%)	Mixed Methods Studies		2 (4.4%)	[57,70]
	Building Industry Reports		3 (6.7%)	[39,72,92]
Qualitative Descriptive Studies 4 (8.9%)	Qualitative Descriptive Studies		3 (6.7%)	[76,87,88]
	Government Inquiry Report		1 (2.2%)	[41]
Study quality appraisal	Authority, Accuracy, Coverage, Objectivity, Date, Significance Checklist (AACODS) *n* = 16	*n* (%)	*n* (%)	Mixed Methods Assessment Tool (MMAT) *n* = 45
	*			
	**	1 (6.3%)	2 (4.4%)	*
	***		1 (2.2%)	**
	****		6 (13.3%)	***
	*****	1 (6.3%)	15 (33.3%)	****
	******	14 (87.5%)	21 (46.7%)	*****

Notes: ****** = 6 questions answered “Yes”, ***** = 5 questions answered “Yes”, **** = 4 questions answered “Yes”, *** = 3 questions answered “Yes”, ** = 2 questions answered “Yes”, * = 1 question answered “Yes”. The AACODS checklist had a total of 6 questions. The MMAT assessment tool had a total of 5 questions.

**Table 5:** Profile of the main study topics within the included studies.

Study Topics	Number (*n* = 45) *n* (%)	Subtopics	Ref
Building characteristics	19 (42.2%)	Indoor biological data	[66,67,68,69,74,81]
		Housing survey data	[60,91]
		Housing defects and “root cause”	[39,42,72,73,82,92]
		Indoor mould intervention	[70,71,84,85,86]
Health	14 (31.1%)	Asthma, allergy, respiratory	[40,62,63,64,79,80,89]
		Hypersensitivity pneumonitis	[93]
		Allergic alveolitis	[75]
		Other	[41,59,65,90,94]
Housing conditions and socio-economic factors	9 (20%)	Energy use and health	[78]
		Health	[76,77,87,88]
		Tenure	[47,48,58,61]
COVID-19 insights and housing conditions	2 (4.4%)	Mental health	[57]
		Renting	[46]
Occupant behaviours	1 (2.2%)	Hygiene practices and health	[83]

**Table 6:** Climatical distribution of included studies reporting housing conditions or building characteristics by climate zones.

Coverage of Climate Zones	Housing Conditions (*n* = 12)		Building Characteristics (*n* = 18)	
	*n* (%)	Ref	*n* (%)	Ref
All climate zones			1 (5.6%)	[39]
1—High humidity summer, warm winter				
2—Warm humid summer, mild winter				
3—Hot dry summer, warm winter				
4—Hot dry summer, cool winter				
5—Warm temperate			1 (5.6%)	[75]
5/6—Warm/mild temperate	2 (16.7%)	[77,78]	3 (16.7%)	[72,73,74]
6—Mild temperate	3 (25%)	[48,61,76]	6 (33.3%)	[40,65,66,67,68,69]
7—Cool temperate			3 (16.7%)	[42,81,82]
8—Alpine				
Unspecified study locations	7 (58.3%)	[46,47,57,58,87,88,91]	4 (22.2%)	[41,60,90,92]

**Table 7:** Relative humidity (RH) levels and risk of indoor mould/mildew/fungi concentrations.

Categories	Climate Zone	Risk Factors for Residential Indoor Mould/Mildew/Fungi	Level of Association
Indoor conditions	5/6	Hot walls compared to cooler room [73]	Y
	5/6	Bedroom relative humidity levels RH > 80% [73]	Y
	6	High indoor humidity RH > 60% [40]	Y *
	6	RH equal > 70% [69]	Y *

Notes: Y = Positive association reported, Y * = Significant association (*p* < 0.05).

**Table 8:** Building characteristics and risk factors reported for indoor mould/mildew/increased fungi concentrations.

Categories	Data Before 2002	Data After 2003	Risk Factors for Residential Indoor Mould/Mildew/Fungi	Level of Association
Housing conditions				
Housing conditions	x		Poor housing conditions [75]	Y *
	x		Age of home >20 years (1992) [69], >10 years (1994) [60], >70 years (in 1991) [75]	Y *, Y, Y *
	x	x	Leaking roof/ceiling [60], water intrusion [40,69], leaks [73]	Y, Y *, PY
	x		Water-damaged/collapsing wooden floorboards [75], cracks in cladding [40]	Y *, Y *
Building characteristics—Building, design, construction				
Construction		x	Exposing building materials to moisture during construction [41]	PY
		x	Building defects (various water/moisture/waterproofing related) [41,72,92]	PY, Y
Building envelope		x	Air-tightness in buildings, surface/interstitial condensation, thermal bridging, non-breathable wall wraps/foil wraps, unventilated walls [39,41,42,82,113,114,115]	PY, Y
		x	Use of timber framing and/or gypsum board [41]	PY
		x	External walls adjoining unheated spaces [42,82,113,114,115]	Y
Roof		x	Blocked gutters/incorrect gutter installation [41]	PY
Walls	x		Brick veneer [60]	Y
	x		Double brick [60,67]	Y *, Y
Foundation	x		Stumps [40]	Y *
Drainage		x	Inappropriate external drainage [73]	PY
Insulation	x	x	Limited or poorly installed insulation [49]	Y *, Y
Building layout	x		Higher number of bedrooms or bathrooms [90]	Y
	x		Airflow from bathrooms towards bedrooms [65,90]	Y
	x		Inadequate building orientation and lack of breezes [65,90]	Y
Windows		x	Single glazed windows [70]	Y
		x	Poorly ventilated areas behind curtains [70]	Y
		x	Lack of natural light [73]	PY
	x		Limited ventilation through open windows [40]	Y *
Cooling/heating		x	Split-system air-conditioning units [41,84]	Y, PY
	x		No solid fuel fire [67]	Y *
	x		Cold bedrooms [40]	Y *
Ventilation/air flow	x	x	Inadequate ventilation [41,73,83,92]	PY, Y, Y *
	x		No bedroom ceiling fan, no kitchen exhaust fan, few extractor fans in wet areas [67]	Y *
non-structural		x	Carpets without professional cleaning [85]	Y
	x		Old carpets: 5 years or older [67]	Y *

Notes: PY = Perceived association, Y = Positive association reported, Y * = Significant association (*p* < 0.05).

**Table 9:** Occupant behaviours and risk of indoor mould/mildew/fungi concentrations.

Categories	Data Before 2002	Data After 2003	Risk Factors for Residential Indoor Mould/Mildew/Fungi	Level of Association
Occupant behaviours				
Ventilation	x		Windows left open [67]	Y *
		x	Occupant unaware of their behaviours with condensation [39]	PY
		x	Occupant reluctant to open windows and doors due to energy costs [39]	PY
	x		Infrequent use of opening windows [40], infrequent natural ventilation [67]	Y *
	x		Lack of opening windows [40]	Y *
Cleaning		x	Unclean plastic seals on a dishwasher doors [71]	Y *
	x		Failure to remove indoor mould growth [40]	Y *
	x		Homes cleaned less [83], vacuuming > 1 week ago [67]	Y *
Pets	x		Presence of 1 cat or presence of 1 dog [67]	Y *

Notes: PY = Perceived association, Y = Positive association reported, Y * = Significant association (*p* < 0.05).

**Table 10:** Findings of symptom outcomes reported in relation to dampness/mould/mildew/fungi indicators.

Symptom/Illness	Sufficient Evidence for an Association by World Health Organization (WHO) [1]	Association	(*n* = 45)
Asthma	Asthma in children [40]	Y *	12 (26.7%)
	Asthma [65,90]	Qual	
	Current asthma [79]	Y **	
	Greater odds for an asthma attack in the last 12 months [62]	Y *	
	Increase in Peak Flow Variability (PFV) in asthmatics sensitised to fungi [63]	Y *	
	Exacerbation of asthma [76]	Qual	
Wheeze	Wheeze [79]	Y **	
	Increase in wheeze [62]	Y	
Cough	Cough [65,90]	Qual	
Respiratory	Acute respiratory illness with cough (ARIwC) in children [89]	Y	
	Respiratory symptoms in children [40]	Y *	
	Respiratory problems/conditions [76,87]	Qual	
	Nocturnal chest tightness [79]	Y **	
	Increased bronchial hyperreactivity (BHR) [64]	Y *	
Clinical	Domestic allergic alveolitis [75]	Y *	
	Hypersensitivity pneumonitis [93]	Y	
	**Other symptom(s)/illness presentation**		
Allergy	Allergy in children [40]	Y *	7 (15.6%)
	Increase in allergy to fungi [62]	Y	
	Protective—Lower risk of allergy to fungi [64]	Y	
	Pollen and dust mite allergy [90]	Qual	
Atopy	Atopy in children [40]	Y *	
	Increase in atopy [62]	Y *	
Gastrointestinal	Gastrointestinal infections in children [77]	Y *	
Mood/depression	Depression [57]	Y	
	Sadness/depression [87]	Qual	
Pain	Joint pain [87,90]	Qual	
	**Multiple-symptom presentation**		
Comorbidity	Biotoxin illness reported with multiple chemical sensitivity (MCS) [94]	Y	5 (11.1%)
	Biotoxin illness reported with tick-borne illness [94]	Y	
ME/CFS	Moulds as a trigger for myalgic encephalomyelitis/chronic fatigue syndrome (ME/CFS) [59]	Y	
	Biotoxin illness reported with ME/CFS [94]	Y	
Multiple-symptom presentation	Chronic fatigue, pain, memory and concentration problems, disorientation, insomnia, gastrointestinal issues, sinus issues, fever, headaches and respiratory issues [41]	Qual	
	Fatigue, bronchial complaints, hay fever, headaches, hyperactivity, hypersensitivity or allergy, mood change, sensitivity to foods, water and textiles, sinus complaints, loss of sense of smell, pollen and dust mite allergy, skin complaints (eczema, itching, inflammation) [65,90]	Qual	

Notes: Qual = Perceived association, Y = Positive association reported, Y * = Significant association (*p* < 0.05), Y ** = Significant association (*p* < 0.001).

## Supplementary Materials

The following are available online at https://www.mdpi.com/article/10.3390/ijerph19031854/s1: Table S1: Search strings with Boolean operators used for each database search; Table S2: MMAT Quality Ratings for Included Studies—Qualitative Descriptive Studies; Table S3: MMAT Quality Ratings for Included Studies—Quantitative Non-randomised Studies; Table S4: MMAT Quality Ratings for Included Studies—Quantitative Descriptive Studies; Table S5: MMAT Quality Ratings for Included Studies—Mixed Method Studies; Table S6: AACODS Quality Ratings for Included Studies—Grey Literature Only; Table S7: Summary of Included Studies by Theme.
