# Supplementary material for: Prevalence, Risk Factors and Impacts Related to Mould-Affected Housing: An Australian Integrative Review
Source: Int J Environ Res Public Health. 2022 Feb 7;19(3):1854. doi: 10.3390/ijerph19031854 (PMC8835129; doi:10.3390/ijerph19031854)
Supplement: Supplementary file 1 [file ijerph-19-01854-s001.zip › ijerph-1547900-supplementary.pdf]

Table S1: Search strings with Boolean operators used for each database search.

Themes	Search Strings for PubMed	Search Strings for Embase
Place / Mould / Housing	(australia[MeSH terms] OR australia[Title/Abstract]) AND (damp*[Title/Abstract] OR mould[Title/Abstract] OR fungi[Title/Abstract] OR mildew[Title/Abstract] OR condensation[Title/Abstract] OR flood*[Title/Abstract] OR "Water damage"[Title/Abstract] OR "Water-damage"[Title/Abstract]) AND (hous*[Title/Abstract] OR indoor[Title/Abstract] OR home*[Title/Abstract] OR dwelling*[Title/Abstract] OR residen*[Title/Abstract] OR building*[Title/Abstract])	#1 Australia:ti,ab
		#2 AND (damp*:ti,ab OR mould:ti,ab OR fungi:ti,ab OR mildew:ti,ab OR condensation:ti,ab OR flood*:ti,ab OR 'Water damage':ti,ab OR 'Water-damage':ti,ab)
		#3 AND (hous*:ti,ab OR indoor:ti,ab OR home*:ti,ab OR dwelling*:ti,ab OR residen*:ti,ab OR building*:ti,ab)
		#1 AND #2 AND #3
Place / Mould / Housing / Health & Wellbeing	(australia[MeSH terms] OR australia[Title/Abstract]) AND (damp*[Title/Abstract] OR mould[Title/Abstract] OR fungi[Title/Abstract] OR mildew[Title/Abstract] OR condensation[Title/Abstract] OR flood*[Title/Abstract] OR "Water damage"[Title/Abstract] OR "Water-damage"[Title/Abstract]) AND (hous*[Title/Abstract] OR indoor[Title/Abstract] OR home*[Title/Abstract] OR dwelling*[Title/Abstract] OR residen*[Title/Abstract] OR building*[Title/Abstract]) AND (health[Title/Abstract] OR wellbeing[Title/Abstract] OR well-being[Title/Abstract])	#1 AND #2 AND #3 AND #5
		#5 AND (health:ti,ab OR wellbeing:ti,ab OR well-being:ti,ab)
Place / Mould / Housing / Risk & Impact	(australia[MeSH terms] OR australia[Title/Abstract]) AND (damp*[Title/Abstract] OR mould[Title/Abstract] OR fungi[Title/Abstract] OR mildew[Title/Abstract] OR condensation[Title/Abstract] OR flood*[Title/Abstract] OR "Water damage"[Title/Abstract] OR "Water-damage"[Title/Abstract]) AND (hous*[Title/Abstract] OR indoor[Title/Abstract] OR home*[Title/Abstract] OR dwelling*[Title/Abstract] OR residen*[Title/Abstract] OR building*[Title/Abstract]) AND (cost*[Title/Abstract] OR impact*[Title/Abstract] OR hardship*[Title/Abstract] OR Economic*[Title/Abstract] OR Financial[Title/Abstract] OR risk*[Title/Abstract] OR perception*[Title/Abstract])	#1 AND #2 AND #3 AND #7
		#7 AND (cost*:ti,ab OR impact*:ti,ab OR hardship*:ti,ab OR Economic*:ti,ab OR Financial:ti,ab OR risk*:ti,ab OR perception*:ti,ab)
Place / Mould / Housing / Health, Wellbeing, Risk & Impact	(australia[MeSH terms] OR australia[Title/Abstract]) AND (damp*[Title/Abstract] OR mould[Title/Abstract] OR fungi[Title/Abstract] OR mildew[Title/Abstract] OR condensation[Title/Abstract] OR flood*[Title/Abstract] OR "Water damage"[Title/Abstract] OR "Water-damage"[Title/Abstract]) AND (hous*[Title/Abstract] OR indoor[Title/Abstract] OR home*[Title/Abstract] OR dwelling*[Title/Abstract] OR residen*[Title/Abstract] OR building*[Title/Abstract]) AND (health[Title/Abstract] OR wellbeing[Title/Abstract] OR well-being[Title/Abstract]) AND (cost*[Title/Abstract] OR impact*[Title/Abstract] OR hardship*[Title/Abstract] OR Economic*[Title/Abstract] OR Financial[Title/Abstract] OR risk*[Title/Abstract] OR perception*[Title/Abstract])	#1 AND #2 AND #3 AND #5 AND #7

Table S1. Search strings with Boolean operators used for each database search (continued).

Themes	Search Strings for Scopus	Search Strings for Science Direct[1]
Place / Mould / Housing	(TITLE-ABS-KEY (australia) AND TITLE-ABS-KEY (damp* OR mould OR fungi OR mildew OR condensation OR flood* OR "Water damage" OR "Water-damage") AND TITLE-ABS-KEY (hous* OR indoor OR home* OR dwelling* OR residen* OR building*))	Australia AND (damp OR mould OR fungi OR mildew OR condensation OR flood OR "Water damage") AND house
		Australia AND (damp OR mould OR fungi OR mildew OR condensation OR flood OR "Water damage") AND housing
		Australia AND (damp OR mould OR fungi OR mildew OR condensation OR flood OR "Water damage") AND indoor
		Australia AND (damp OR mould OR fungi OR mildew OR condensation OR flood OR "Water damage") AND home
		Australia AND (damp OR mould OR fungi OR mildew OR condensation OR flood OR "Water damage") AND dwelling
		Australia AND (damp OR mould OR fungi OR mildew OR condensation OR flood OR "Water damage") AND residence
		Australia AND (damp OR mould OR fungi OR mildew OR condensation OR flood OR "Water damage") AND residential
		Australia AND (damp OR mould OR fungi OR mildew OR condensation OR flood OR "Water damage") AND building
Place / Mould / Housing / Health & Wellbeing	(TITLE-ABS-KEY (australia) AND TITLE-ABS-KEY (damp* OR mould OR fungi OR mildew OR condensation OR flood* OR "Water damage" OR "Water-damage") AND TITLE-ABS-KEY (hous* OR indoor OR home* OR dwelling* OR residen* OR building*) AND TITLE-ABS-KEY (health OR wellbeing OR well-being))	Australia AND residential AND housing AND mould AND risk AND impact AND health
		Australia AND (damp OR mould OR flood OR "Water damage") AND (house OR home) AND (health OR well-being)
		Australia AND (damp OR mould OR flood OR "Water damage") AND (dwelling OR residence) AND (health OR well-being)
		Australia AND (damp OR mould OR flood OR "Water damage") AND (building OR residential) AND (health OR well-being)
Place / Mould / Housing / Risk & Impact	(TITLE-ABS-KEY (australia) AND TITLE-ABS-KEY (damp* OR mould OR fungi OR mildew OR condensation OR flood* OR "Water damage" OR "Water-damage") AND TITLE-ABS-KEY (hous* OR indoor OR home* OR dwelling* OR residen* OR building*) AND TITLE-ABS-KEY (cost* OR impact* OR hardship* OR economic* OR financial OR risk* OR perception*))	Australia AND dwelling AND residential AND housing AND mould AND risk AND impact AND health
		Australia AND (damp OR mould OR flood OR "Water damage") AND (house OR home) AND (cost OR impact)
		Australia AND (damp OR mould OR flood OR "Water damage") AND (housing OR indoor) AND (cost OR impact)
Place / Mould / Housing / Health, Wellbeing, Risk & Impact	(TITLE-ABS-KEY (australia) AND TITLE-ABS-KEY (damp* OR mould OR fungi OR mildew OR condensation OR flood* OR "Water damage" OR "Water-damage") AND TITLE-ABS-KEY (hous* OR indoor OR home* OR dwelling* OR residen* OR building*) AND TITLE-ABS-KEY (health OR wellbeing OR well-being) AND TITLE-ABS-KEY (cost* OR impact* OR hardship* OR economic* OR financial OR risk* OR perception*))	Included above

[1] Science Direct has a limit on the number of key word fields and searches.

Table S2: MMAT Quality Ratings for Included Studies – Qualitative Descriptive Studies.

**Rating Criteria**	**Author(s)**			
	(Ziersch, Walsh, et al., 2017)	(Ziersch, Due, et al., 2017)	(Andersen et al., 2016)	(Commonwealth of Australia. House of Representatives Standing Committee on Health Aged Care and Sport, 2018)
1. Is the qualitative approach appropriate to answer the research question?	Y	Y	Y	Y
2. Are the qualitative data collection methods adequate to address the research question?	Y	Y	Y	Y
3. Are the findings adequately derived from the data?	Y	Y	Y	Y
4. Is the interpretation of results sufficiently substantiated by data?	Y	Y	Y	Y
5. Is there coherence between qualitative data sources, collection, analysis and interpretation?	Y	Y	Y	Y

Notes: Grey background indicates that the study was not published in an academic journal/publication. Y = Yes, N = No, U = Unknown, N/a = Not applicable

Table S3: MMAT Quality Ratings for Included Studies – Quantitative Non-randomized Studies.

Rating Criteria	Author(s)																	
	(Andersen, Skinner, et al., 2018)	(Andersen, Williamson, et al., 2018)	(Zhang et al., 2005)	(Hall et al., 2017)	(Mészáros et al., 2014)	(Matheson et al., 2005)	(Dharmage et al., 2002)	(Dharmage et al., 2001)	(Ponsonby et al., 2000)	(Mihrshahi et al., 2002)	(Dharmage, Bailey, Raven, Cheng, et al., 1999)	(Dharmage, Bailey, Raven, Mitakakis, et al., 1999)	(Couper et al., 1998)	(Garrett, Hooper, et al., 1998)	(Godish et al., 1996)	(Midson et al., 2012)	(C. Cheong et al., 2007)	(C. D. Cheong et al., 2004)
1. Are the participants representative of the target population?	Y	N	Y	Y	Y	Y	Y	Y	Y	Y	Y	Y	Y	Y	Y	N	Y	Y
2. Are measurements appropriate regarding both the outcome and intervention (or exposure)?	Y	Y	Y	Y	Y	Y	Y	Y	Y	Y	Y	Y	Y	Y	Y	N	Y	Y
3. Are there complete outcome data?	Y	Y	Y	Y	Y	Y	Y	Y	Y	Y	Y	Y	Y	Y	Y	N	Y	Y
4. Are the confounders accounted for in the design and analysis?	Y	Y	Y	N	Y	Y	Y	Y	Y	N	Y	Y	Y	N	N	N	N	Y
5. During the study period, is the intervention administered (or exposure occurred) as intended?	N	Y	N	Y	Y	N	Y	Y	Y	Y	Y	Y	Y	Y	Y	Y	Y	Y

Notes: Grey background indicates that the study was not published in an academic journal/publication. Y = Yes, N = No, U = Unknown, N/a = Not applicable

Table S4: MMAT Quality Ratings for Included Studies – Quantitative Descriptive Studies.

Rating Criteria	Author(s)																	
	(Baker & Daniel, 2020)	(Haddad et al., 2019)	(Choice et al., 2018)	(Choice & National Shelter, 2016)	(Victorian Council of Social Service (VCOSS)., 2010)	(Saltos et al., 1982)	(Martin, 2019)	(S. C. Johnston et al., 2016)	(Robertson, 2001)	(Garrett, Rayment, et al., 1998)	(Robertson, 1992)	(Bryant & Rogers, 1991)	(Baker et al., 2018)	(Trewin, 1999)	(Kempton et al., 2021)	(Law & Dewsbury, 2018)	(Dewsbury, Law, & Henderson, 2016d)	(Zalar et al., 2011)
1. Is the sampling strategy relevant to address the research question?	Y	Y	Y	Y	Y	Y	Y	Y	Y	Y	Y	Y	Y	Y	Y	Y	Y	Y
2. Is the sample representative of the target population?	Y	N	Y	Y	N	N/a	N	N	N	Y	Y	Y	N	Y	Y	N/a	N/a	N/a
3. Are the measurements appropriate?	Y	N	Y	Y	Y	Y	N	Y	Y	Y	Y	Y	Y	Y	Y	Y	Y	Y
4. Is the risk of nonresponse bias low?	Y	U	U	U	N/a	Y	Y	N	Y	N	Y	Y	Y	Y	N/a	Y	Y	N/a
5. Is the statistical analysis appropriate to answer the research question?	Y	N	Y	Y	N	N/a	Y	Y	Y	Y	Y	Y	Y	Y	Y	N/a	N/a	Y

Notes: Grey background indicates that the study was not published in an academic journal/publication. Y = Yes, N = No, U = Unknown, N/a = Not applicable

Table S5: MMAT Quality Ratings for Included Studies – Mixed Method Studies.

Rating Criteria	Author(s)				
	(Bower et al., 2021)	(N. Johnston & Reid, 2019)	(Crommelin et al., 2021)	(Dewsbury, Law, Potgieter, et al., 2016)	(Willand et al., 2019)
1. Is there an adequate rationale for using a mixed methods design to address the research question?	Y	Y	Y	Y	Y
2. Are the different components of the study effectively integrated to answer the research question?	Y	Y	Y	Y	Y
3. Are the outputs of the integration of qualitative and quantitative components adequately interpreted?	Y	Y	Y	Y	Y
4. Are divergences and inconsistencies between quantitative and qualitative results adequately addressed?	Y	Y	Y	Y	Y
5. Do the different components of the study adhere to the quality criteria of each tradition of the methods involved?	Y	Y	Y	Y	Y

Notes: Grey background indicates that the study was not published in an academic journal/publication. Y = Yes, N = No, U = Unknown, N/a = Not applicable

Table S6: AACODS Quality Ratings for Included Studies – Grey Literature only.

Rating Criteria	Author(s)															
	(Baker & Daniel, 2020)	(Haddad et al., 2019)	(Choice et al., 2018)	(Ziersch, Due, et al., 2017)	(Choice & National Shelter, 2016)	(Victorian Council of Social Service (VCOSS)., 2010)	(Martin, 2019)	(Commonwealth of Australia. House of Representatives Standing Committee on Health Aged Care and Sport, 2018)	(Baker et al., 2018)	(Trewin, 1999)	(N. Johnston & Reid, 2019)	(Crommelin et al., 2021)	(Dewsbury, Law, Potgieter, et al., 2016)	(Dewsbury, Law, & Henderson, 2016d)	(Midson et al., 2012)	(C. Cheong et al., 2007)
1. Authority:	Y	Y	Y*	Y	Y*	Y*	Y*	Y**	Y	Y**	Y	Y	Y	Y	N	Y
2. Accuracy:	Y	Y	Y	Y	Y	Y	Y	Y	Y	Y	Y	Y	Y	Y	N	Y
3. Coverage:	Y	Y	Y	Y	Y	Y	Y	Y	Y	Y	Y	Y	Y	Y	N	Y
4. Objectivity:	Y	Y	Y	Y	Y	Y	Y	Y	Y	Y	Y	Y	Y	Y	N	N
5. Date:	Y	Y	Y	Y	Y	Y	Y	Y	Y	Y	Y	Y	Y	Y	Y	Y
6. Significance:	Y	Y	Y	Y	Y	Y	Y	Y	Y	Y	Y	Y	Y	Y	Y	Y

Notes: Grey background indicates that the study was not published in an academic journal/publication. Y = Yes – University/research institutes/industry & university partnership, Y* = Yes – Advocacy/private business, Y** = Government, N = No, U = Unknown

AACODS Questions (Tyndall, 2010)

1.	Authority:	Individual author: •Associated with a reputable organisation? • Professional qualifications or considerable experience? • Produced/published other work (grey/black) in the field? • Recognised expert, identified in other sources? • Cited by others? (use Google Scholar as a quick check) • Higher degree student under "expert" supervision? Organisation or group: • Is the organisation reputable? (e.g. W.H.O) • Is the organisation an authority in the field? In all cases: • Does the item have a detailed reference list or bibliography?
2.	Accuracy:	• Does the item have a clearly stated aim or brief? • Is so, is this met? • Does it have a stated methodology? • If so, is it adhered to? • Has it been peer-reviewed? • Has it been edited by a reputable authority? • Supported by authoritative, documented references or credible sources? • Is it representative of work in the field? • If No, is it a valid counterbalance? • Is any data collection explicit and appropriate for the research? • If item is secondary material (e.g. a policy brief of a technical report) refer to the original. Is it an accurate, unbiased interpretation or analysis?
3.	Coverage:	All items have parameters which define their content coverage. These limits might mean that a work refers to a particular population group, or that it excluded certain types of publication. A report could be designed to answer a particular question, or be based on statistics from a particular survey. • Are any limits clearly stated?
4.	Objectivity:	It is important to identify bias, particularly if it is unstated or unacknowledged. • Opinion, expert or otherwise, is still opinion: is the author's standpoint clear? • Does the work seem to be balanced in presentation?
5.	Date:	For the item to inform your research, it needs to have a date that confirms relevance • Does the item have a clearly stated date related to content? No easily discernible date is a strong concern. • If no date is given, but can be closely ascertained, is there a valid reason for its absence? • Check the bibliography: have key contemporary material been included?
6.	Significance:	This is a value judgment of the item, in the context of the relevant research area • Is the item meaningful? (this incorporates feasibility, utility and relevance) • Does it add context? • Does it enrich or add something unique to the research? • Does it strengthen or refute a current position? • Would the research area be lesser without it? • Is it integral, representative, typical? • Does it have impact? (in the sense of influencing the work or behavior of others)

Table S7: Summary of Included Studies by Theme.

Author(s) & Year / Data Year	Location / Housing Types	Study Group / Tenure	Study Type	Risk Factors for mould/mildew/fungi	Association	Key findings / Impacts / Prevalence
Theme – COVID-19 insights & housing structural conditions						
'Trapped', 'anxious' and 'traumatised': COVID-19 intensified the impact of housing inequality on Australians' mental health (Bower et al., 2021)<br><br>**Data Year:**<br>2020	**Location:**<br>National / unknown<br><br>**Housing Types:**<br>Mixed housing types	Adults<br>n=2,065<br><br>Mixed Tenure	MMR<br><br>Cross sectional<br><br>SRQ & IQ<br>QUALo	**Poor housing conditions including;**<br>Mould, cracks, dampness	Y	**Health impacts from poor housing conditions:**<br>Depression.<br><br>For every major structural physical issue (mould, cracks, damp) the likelihood of Depression increased by 14%. Note: associations were no longer significant when covariates were accounted for.<br><br>**Prevalence:**<br>21% (n=380) reported between 2 and 9 major structural building problems<br>17.8% reported had 1 major structural building problem<br><br>**Key findings:**<br>Increased time at home had both protective and negative consequences.
Rental Insights A COVID-19 Collection AHUI<br><br>(Baker & Daniel, 2020)<br><br>**Data Year:**<br>2020	**Location:**<br>National / unknown<br><br>**Housing Types:**<br>Mixed	Households<br>N=14,486<br><br>Rental (Private rental / public & community housing)	QUAN<br><br>Prevalence study<br><br>SRQ<br>Dataset	**Poor housing conditions including;**<br>Mould, damp, cold, cracks<br><br>**Socioeconomic circumstance:**<br>Unaffordable housing<br><br>Younger households (18–49 years old) compared households aged over 50 years<br>Couples with children<br>Families with children<br>Single parent-headed families		**Health impacts from poor housing conditions:**<br>'Poor' general health related to Approx. 37% mould<br>'Poor' general health related to Approx. 36% cold<br>'Poor' general health related to Approx. 32.5% damp<br><br>People reporting Asthma had, 27% cold, 35% mould & 28% damp. These were higher than the broader sample.<br><br>A few quotes – 'Allergies, bronchitis and breathing difficulties from living in damp, wet, cold and mouldy housing'.<br><br>**Prevalence:**<br>40% of rental dwellings reported problems with cracks in walls and floors<br>27% of rental dwellings reported problems with mould<br>21% of rental dwellings reported problems with dampness<br>23% of rental dwellings reported not being able to keep warm in their home during cold weather<br>69–78% in 'Very Poor' quality houses.<br><br>**Key findings:**

						Widespread problems of cold, mould and damp, and close to 50% families with children in social housing reported multiple problem conditions (including dampness / mould)
Theme – Housing structural conditions, socioeconomic circumstance & health						
An extensive study on the relationship between energy use, indoor thermal comfort, and health in social housing: the case of the New South Wales, Australia<br>(Haddad et al., 2019)<br><br>**Data Year:**<br>2018 / 2019	**Location:**<br>Sydney metro, NSW<br><br>Climate Zone 5/6<br><br>**Housing Types:**<br>Mixed	Households N=106<br>Residents n=109<br><br>Social housing (public, community & affordable housing)	QUAN Cohort Study<br><br>IAS<br>Survey<br>Inspector	**Poor housing conditions including;**<br>Mould<br><br>**Socioeconomic circumstance:**<br>People living in social housing		**Health impacts from poor housing conditions:**<br>28% psychological disorders (including depression)<br>32% allergies, 54% reported specific health problems (airway 20% / blood disease 20%) or pain<br>Out of 42 children 26% reported allergic reactions<br><br>**Prevalence:**<br>42% of dwellings had problems with mould and condensation<br>40% mould in the bedrooms<br>35% wet areas (bathroom, laundry, kitchen)<br>17% in living areas, 5% in all rooms<br><br>**Key findings:**<br>Summer indoor temps exceeding 39.8c, participants spent an average of 18 hrs at home per day, problems with mould and condensation and health impacts from poor housing quality were common amongst people living in social housing.
Disrupted: The consumer experience of renting in Australia<br>(Choice et al., 2018)<br><br>**Data Year:**<br>2018	**Location:**<br>National<br><br>**Housing Types:**<br>Mixed	Adults n=1547<br><br>Rental	Prevalence study<br><br>SRQ	**Poor housing conditions including:**<br>Mould<br><br>**Socioeconomic circumstance:**<br>Tenants in private rental market<br>Families with children<br><br>**Building characteristics:**<br>Poor ventilation<br>Difficulty to keep property warm<br>Difficulty to keep property cool	 PY	**Prevalence:**<br>51% of people who rent living in a home that is need of repairs<br>33% renters have experienced problems with mould in bathroom<br>14% renters have experienced problems with mould in bedrooms<br>35% all people who rent reporting mould in their bathrooms and 20% reporting mould in their bedrooms<br><br>**Key findings:**<br>Almost 2 in 5 (39%) families with children have reported issues with mould in the bathroom, compared with 29% of other people who rent. NSW has the highest reported incidence of mould. Mould that is difficult to remove or that reappears remains a constant issue for people who rent across Australia.
Exploring the Relationship between Housing and Health for Refugees and Asylum Seekers in South Australia<br>(Ziersch, Walsh, et al., 2017)<br><br>**Data Year:**<br><2017	**Location:**<br>SA<br><br>Climate Zone unclear<br><br>**Housing Types:**	Adults from refugee or asylum-seeking backgrounds (n=50 (28 refugee & 22	Qualitative Descriptive study<br><br>QUALi	**Poor housing conditions including:**<br>Mould,<br>Cold and damp<br>Lack of heating and/or cooling,<br>Leaking pipes,<br>Leaking ceilings,<br>Peeling paint<br>Overcrowding	 PY<br>PY	**Health impacts from poor housing conditions:**<br>Cold, damp and mouldy housing was identified to be one of the most significant factors reported across the interviews perceived to be causing a range of physical and mental health issues, including:<br>Respiratory problems<br>Sadness / depression<br>Joint pain.

	Unknown	asylum seekers)) <br>Rental		Broken amenities <br>**Socioeconomic circumstance:** Refugee status		**Key findings:** Housing affordability, poor housing conditions, housing layout played key roles in the perceived health of the occupants. Poor housing conditions were perceived to be associated with housing affordability.
Belonging Begins at Home: Housing, social inclusion and health and wellbeing for people from refugee and asylum seeking backgrounds (Ziersch, Due, et al., 2017) <br>**Data Year:** 2015-2017	**Location:** SA <br>Climate Zone unclear <br>**Housing Types:** Unknown	Adults from refugee or asylum seeking backgrounds n=423	Qualitative descriptive study <br>SRQ QUALi VI	**Poor housing conditions including:** Mouldy Cold and damp Poor dwelling condition Absence of functioning heating and cooling	PY	**Health impacts from poor housing conditions:** 80% of people thought that housing had an impact on their health and wellbeing to some extent, with 25% reported that their housing affected health a great deal <br>**Key findings:** Living in poor housing condition perceived to impact health and wellbeing, and no heating and cooling affected mood and exacerbated back and joint pain.
Unsettled: Life in Australia's private rental market (Choice & National Shelter, 2016) <br>**Data Year:** 2016	**Location:** National <br>**Housing Types:** Mixed	Adults n=1005 <br>Private rental	Prevalence study <br>SRQ	**Poor housing conditions including:** Mould <br>**Socioeconomic circumstance:** Tenants in private rental market		**Prevalence:** 21% experienced Leaks or flooding 20% experienced mould that is difficult to remove or reappears 18% difficulty to keep property warm or cold <br>**Key findings:** 8% of people were living in properties that require urgent repairs, with women only households, people on month-to month tenancy agreements and renters on low income.
Housing conditions associated with recurrent gastrointestinal infection in urban Aboriginal children in NSW, Australia findings from SEARCH. (Andersen, Skinner, et al., 2018) <br>**Data Year:** 2007 to 2011	**Location:** NSW, Sydney (Urban) <br>Climate Zone 5/6 <br>**Housing Types:** Unknown	Children n=1398 from indigenous backgrounds	QUAN Cohort Study	**Poor housing conditions including:** Major structural problems Damp or mildew Overcrowding Could not be kept warm enough Vermin <br>**Socioeconomic circumstance:** Urban indigenous children in NSW	Y* <br>Y* <br>Y*	**Health impacts from poor housing conditions:** Recurrent gastrointestinal infection ever was associated with damp or mildew, major structural problems and carer reporting of feeling crowded. <br>Living in a home with major structural problems, vermin problems or that could not be kept warm enough in winter was associated with recurrent gastrointestinal infection treated in the past month <br>Children in homes with three or more housing problems were more than twice as likely to have ever had recurrent gastrointestinal infection as those in homes with 0–2 housing problems. <br>**Key findings:** The number of housing condition problems were associated with increasing risk of recurrent gastrointestinal infection in children.
Decent not dodgy (Victorian Council of Social Service (VCOSS), 2010)	**Location:** VIC, Melbourne	N=116 <br>Private rental	Prevalence study	**Poor housing conditions including:** Visible and extensive mould Uninhabitable		**Prevalence:** 19% reported visible and extensive mould (majority in the bathroom) 12% uninhabitable

**Data Year:**<br><2010	Climate Zone 6<br><br>**Housing Types:** Mixed		VI<br>BC	No heating<br>Lack of ventilation		10% no heating<br><br>**Key findings:**<br>Lack of ventilation **-** Of the 12 % of properties surveyed, where one or more rooms did not have a window that was able to be opened for ventilation, 53 % had one room with no ventilation, 29 % had two rooms where windows could not be opened and 2 properties surveyed had five rooms where windows could not be opened.
There's a housing crisis going on in Sydney for Aboriginal people _ focus group accounts of housing and perceived associations with health (Andersen et al., 2016)<br><br>**Data Year:**<br>2010	**Location:** NSW, Western Sydney (Urban)<br><br>Climate Zone 6<br><br>**Housing Types:** Mixed	Children & families staff n=38	Qualitative descriptive study<br><br>QUALi<br>SRQ	**Poor housing conditions including:**<br>Mould<br>Damp<br>Broken amenities<br>Leaking rooves<br>Structural problems<br>Faulty plumbing<br>Poor temperature control<br><br>**Socioeconomic circumstance:**<br>Urban housing for indigenous populations	 PY<br>PY	**Health impacts from poor housing conditions:**<br>Perceived associated with the exacerbation of asthma and respiratory conditions from mould and damp.<br><br>**Key findings:**<br>High prevalence of housing problems amongst urban housing for indigenous populations
Housing conditions of urban households with Aboriginal children in NSW Australia: tenure type matters (Andersen, Williamson, et al., 2018)<br><br>**Data Year:**<br>2008 to 2010	**Location:** NSW, Western Sydney (urban)<br><br>Climate Zone 6<br><br>**Housing Types:** Mixed	Caregivers (600), indigenous children (1406) n=600<br><br>Mixed tenure types: Own/mortgage, private rent, social housing	QUAN Cohort Study<br><br>VI	**Poor housing conditions including:**<br>Rising damp<br>Damp/mildew on walls, ceilings, windows<br>Major cracks in walls/floors<br>Wood rot / termite damage<br><br>**Socioeconomic circumstance:**<br>Social housing	 Y*<br>Y**<br>Y*<br>Y*	**Socioeconomic risk:**<br>Social housing<br>Social housing<br>Social housing<br>Social housing<br><br>**Prevalence:**<br>34% of homes reported damp and mildew of those;<br>19% reported rising damp<br>33% reported damp/mildew on walls, ceilings and windows<br><br>**Key findings:**<br>Social housing accounted for 43% of the damp and mildew affected homes, whilst renters 28% and owner/mortgage 13%
Theme – Occupant behaviors & health						
Household hygiene practices in relation to dampness at home and current wheezing and rhino-conjunctivitis among school age children (Zhang et al., 2005)<br><br>**Data Year:**<br>2002	**Location:** WA, Perth Metro<br><br>Climate zone 5<br><br>**Housing**	Children N=997	QUAN Cross sectional<br><br>SRQ	**Risk - occupant behaviours:**<br>Highly cleaned homes<br><br>Less ventilated & homes cleaned less<br>Homes with less ventilation<br>**Protective - occupant behaviours:**<br>Highly natural vented homes<br>Highly cleaned homes	 Y*<br><br>Y*<br>Y*<br><br>Y*<br>Y*	**Key findings with regards health impacts**:<br>Higher prevalence of current wheezing & rhino-conjunctivitis<br>**Key findings with regards to mould:**<br>Visible moulds<br>Damp patches & condensation<br><br><br>Decreased damp patches, condensation and mould

	**Types:**<br>Unknown					Decreased presence of mould in home<br><br>**Prevalence:**<br>28% (n=970) reported having *condensation*<br>23.1% of (n=977) reported having *damp clothes anywhere inside the house*.<br>8.5% (n=972) reported having *damp patches*<br>19.2% (n=866) reported having *visible mould*
Theme – Indoor mould, building characteristics, housing biological data & health						
The incidence and short-term outcomes of acute respiratory illness with cough in children from a socioeconomically disadvantaged urban community in Australia: A community-based prospective cohort study.<br>(Hall et al., 2017)<br><br>**Data Year:**<br>2013 to 2015	**Location:**<br>QLD, Brisbane (urban)<br><br>Climate zone 2<br><br>**Housing Types:**<br>Unknown	Children < 5yrs, n=200<br><br>90% from indigenous backgrounds	QUAN<br>Cohort Study<br><br>SRQ	**Risk factors:**<br>Mould in home	 Y	**Key findings with regards health impacts**:<br>Mould in the house was a predictor of acute respiratory illness with cough (ARIwC) in children.<br><br>The presence of eczema, parent/carer employment status and having an Aboriginal and Torres Strait Islander mother/non-Aboriginal and Torres Strait Islander father was also a predictor of (ARIwC) in children.
Domestic airborne pollutants and asthma and respiratory symptoms in middle age<br>(Mészáros et al., 2014)<br><br>**Data Year:**<br>2004	**Location:**<br>TAS<br><br>Climate zone 7<br><br>**Housing Types:**<br>Unknown	Adults<br>n=5729	QUAN<br>Cohort Study<br><br>SRQ<br>VI	**Risk factors:**<br>Recent mould<br>Recent mould (stratified by atopy & gender)<br>More rooms affected by mould	 Y**<br>Y**<br>Y*	**Key findings with regards health impacts**:<br>Increased risk of current asthma, wheeze, nocturnal chest tightness<br>Increased risk of current non-atopic asthma only in males<br>Increase trend for current asthma, wheeze and nocturnal chest tightness<br>**Protective:**<br>Reverse cycle air-conditioning was negatively associated with doctor diagnose asthma<br>**Prevalence:**<br>48.8% reported that mould was *ever* in the house (*on any home surface*)<br>34.7% reported mould in the home in the last 12 months
Changes in indoor allergen and fungal levels predict changes in asthma activity among young adults<br>(Matheson et al., 2005)<br><br>**Data Year:**<br>Data from 1996 and then in 1998	**Location:**<br>VIC<br>Melb.<br><br>Climate zone 6<br><br>**Housing Types:**<br>Unknown	Young adults<br>N=485<br>N=359	QUAN<br>Cohort Study<br><br>IQ,<br>AS / DS	**Biological risk:**<br>Doubled levels of total fungi<br>Doubled levels of Cladosporium<br><br>Increase in fungal exposure<br>Increase in fungal exposure<br>Increase in fungal exposure<br>Increase in fungal exposure	 Y*<br>Y*<br><br>Y*<br>Y*<br>Y<br>Y	**Key findings with regards health impacts**:<br>53% greater odds of developing atopy<br>52% greater odds of having had an attack of asthma in the last 12 months<br>Onset and persistence of Asthma<br>Onset and increase in atopy<br>Increase in wheeze<br>Increase in allergy to fungi
Mouldy houses influence symptoms of asthma among atopic individuals<br>(Dharmage et al., 2002)	**Location:**<br>VIC<br>Latrobe Valley – Rural	Young adults<br>N=35	QUAN<br>Cohort Study	**Risk factors:**<br>Current visible mould	 Y*	**Key findings with regards health impacts**:<br>Increase in Peak Flow Variability (PFV) in a group of asthmatics sensitized to fungi.<br>**Prevalence:**

**Data Year:**<br>1997/ 98	Climate zone 6<br><br>**Housing Types:**<br>Freestanding houses		SRQ, IQ<br>AS / DS			25.7% had current visible mould in winter<br>14.3% had current visible mould in spring<br>17.1% had current visible mould in summer<br>8.6% had current visible mould in autumn<br>42.9% of participants admitted to observing mould in their bedrooms at the beginning of the study
Current indoor allergen levels of fungi and cats, but not house dust mites, influence allergy and asthma in adults with high dust mite exposure<br>(Dharmage et al., 2001)<br><br>**Data Year:**<br>1996	**Location:**<br>VIC<br>Melbourne<br><br>Climate zone 6<br><br>**Housing Types:**<br>Freestanding houses	Young adults homes<br>N=485	QUAN<br>Cohort Study<br><br>IQ, DS	**Biological risk:**<br>High levels of total airborne fungi<br><br>Current indoor levels of fungi	 Y*<br><br>Y	**Key findings with regards health impacts**:<br>Increased bronchial hyperreactivity (BHR)<br>**Protective:**<br>Lower risk of allergy to fungi<br>**Prevalence of homes:**<br>55% of the houses (n=485) had viable airborne fungal propagules concentrations exceeding 500 CFU/m
The Relation between Infant Indoor Environment and Subsequent Asthma<br>(Ponsonby et al., 2000)<br><br>**Data Year:**<br>From 1995	**Location:**<br>TAS<br><br>Climate zone 7<br><br>**Housing Types:**<br>Unknown	Infants<br>n=863	QUAN<br>Cohort Study<br><br>IQ, HT, VI, RH	**Combination of risk factors:**<br>Mould (observed or reported) and high indoor relative humidity and 6 or more residents in the home.	 Y*	**Biological impact:**<br>High house-dust-mite allergen (Der p 1)<br>**Prevalence:**<br>15.8% of n=855 homes, the mothers reported mould found inside the house (excluding bathroom)<br>4.7% of n=855 homes, the inspector observed mould in the infant's bedroom<br>**Findings:**<br>Poor indoor air quality in an infant's home may play a role in the development of childhood asthma.
Hypersensitivity pneumonitis in a mouldy house<br>(Saltos et al., 1982)<br><br>**Data Year:**<br>1979	**Location:**<br>Unknown<br><br>**Housing Types:**<br>Unknown	Adult / child<br>n=2<br>N=1	Case Report	**Risk factors:**<br>Old house, damp, dust and mould behind wallpapers and under carpets.	 Y	**Key findings with regards health impacts**:<br>Hypersensitivity pneumonitis<br>**Biological findings:**<br>Exposure to thermophilic microorganisms prevalent in their domestic environment. Fungi of the Aspergil/us, Penicil/ium and Cladosporium species were isolated from samples obtained from the walls and floor, but thermophilic actinomycetes were not isolated. Unable to prove causative factors.<br>**Key Findings:**<br>Both patients recovered after moving from this place of residence and medical treatment.
ANRES: A snapshot of living with Environmental Sensitivities in Australia in 2019	**Location:**<br>Unknown	Adults & children<br>n=310	Prevalence study	**Risk factors:**<br>Mould and water damaged buildings	 PY	**Key findings with regards health impacts**:<br>Mould sensitivity reported as a common trigger for Multiple Chemical Sensitivity (MCS)

(Martin, 2019)<br><br>**Data Year:**<br>2019	**Housing Types:**<br>Unknown		SRQ			8.7% of 310 participants (n=27) reported a *biotoxin illness*. Of those reporting a *biotoxin illness* - 25 out of the 27 reported having MCS<br>5 out of the 27 reported Lyme had a *biotoxin illness*. Out of the 27 reported CFS/ME, 18 had *biotoxin illness. Biotoxin illness* was reported to intersect with Lyme, CFS/ME & MCS
Report on the Inquiry into Biotoxin-related Illnesses in Australia (Commonwealth of Australia. House of Representatives Standing Committee on Health Aged Care and Sport, 2018)<br><br>**Data Year:**<br>2018	**Location:**<br>NSW, VIC, QLD, WA + unknown<br><br>**Housing Types:**<br>Mixed	Individual submissions n=114<br><br>Business submissions n=28<br><br>Total n=142	Qualitative descriptive study<br><br>QUALo Government inquiry	**Risk factors:**<br>Presence of mould in homes and/or workplaces<br><br>**Building characteristic risk factors:**<br>Interstitial condensation, air condition systems, air-conditioned at all times, rental homes, gutters installation, inadequate ventilation, inadequate and/or incorrectly installed, waterproofing, lack of timely or appropriate repair or remediation, use of timber framing and/or gypsum board, exposing building materials to moisture during construction; uncovering of 'hidden mould' in wall cavities during renovation works.	PY	**Key findings with regards health impacts**:<br>Anecdotal reports describing the following symptoms that have been attributed to exposure to mould; chronic fatigue, pain, memory and concentration problems, disorientation, insomnia, gastrointestinal issues, sinus issues, fever, headaches and respiratory issues<br><br>Terminology used for illness perceived as assoicated with indoor mould/damp housing inluded; CIRS , CIRS-Water Damaged Building, biotoxin-related illness<br>and mould illness.
Epidemiological characteristics of chronic fatigue syndrome/myalgic encephalomyelitis in Australian patients<br>(S. C. Johnston et al., 2016)<br><br>**Data Year:**<br>2013 to 2015	**Location:**<br>National<br><br>**Housing Types:**<br>Unknown	Adults with CFS/ME n=535	QUAN Cohort Study<br><br>SRQ<br>VI	**Risk factors:**<br>Molds	PY	**Key findings with regards health impacts**:<br>Illness onset trigger for 6.2% (n=10) of patients with CFS/ME (Fukuda criteria) and 9.4% (n=16) of patients with CFS/ME (ICC criteria)

Spatial characteristics of southeast Australian housing linked with allergic complaint<br>(Robertson, 2001)<br><br>**Data Year:**<br>1983 to 94	**Location:**<br>Melbourne<br>VIC, TAS, NSW<br><br>**Housing Types:**<br>Mixed	Homes<br>N=38	QUAN<br>Prevalence study<br><br>VI	**Risk factors**:<br>Dampness faults<br><br>Dampness - which was defined as moisture/condensation related repairs to the home or (exhaust fans, drainage, plumbing leaks etc) OR the replacement of damp-affected materials..<br><br>**Building characteristic risk factors**:<br>Risk of dampness faults may have multiple factors to do with building orientation, microclimate (with regards to wind), adjacencies of rooms / bathroom windows and higher number of bedrooms or bathrooms.	 PY	**Key findings with regards health impacts**:<br>Symptoms included: fatigue, hay fever, headache, mood change, joint pain, sensitivity to foods, water and textiles, disturbed sense of smell, diagnosed asthma, eczema, pollen and dust mite allergy.<br><br>**Prevalence:**<br>55% of the 29 homes in Melbourne had damp faults in the Melbourne-based complaint housing, however, the reporting of visible mould in bathrooms were excluded if cleaned by occupants<br><br>**Recommendations:**<br>Consideration must be given to the orientation of room types in relation to common wind directions to help avoid moisture related issues.
Indoor airborne fungal spores, house dampness and associations with environmental factors and respiratory health in children<br>(Garrett, Rayment, et al., 1998)<br><br>**Data Year:**<br>1994/ 95	**Location:**<br>VIC<br>Latrobe Valley – Rural<br><br>Climate zone 6<br><br>**Housing Types:**<br>Freestanding houses	Children (n=148)<br>Homes<br>N=80	QUAN<br>Cohort Study<br><br>SRQ, IQ, IAS, VI, RH,	**Biological risks:**<br>Indoor exposure to certain fungal genera in winter.<br><br>**Building characteristic risk factors:**<br>Condensation & visible mould growth<br>Bedrooms with cool temp, musty odour, water intrusion, foundation as stumps, cracks in cladding, limited ventilation through open windows, infrequent use of opening windows, few extractor fans in the wet areas, and opening windows only 2-6 or 6-10 months / year<br><br>**Occupant behaviour:**<br>Failure to remove mould growth<br>High indoor humidity RH > 60%<br>Limited (one location) of insulation	 Y*<br><br><br>Y*<br>Y*<br><br><br>Y*<br>Y*<br>Y*	**Key findings with regards health impacts**:<br>Allergy, asthma, atopy and respiratory symptoms. Fungal allergies were more common in winter for children exposed to Cladosporium or Penicillium spores or to a musty odour.<br>**Biological risk factors findings:**<br>Large concentrations of Cladosporium spores<br>Larger total airborne fungal spore concentrations<br><br>Larger total airborne fungal spore concentrations<br>Large concentrations of Penicillium<br>Larger viable spore concentrations<br><br>**Prevalence within homes:**<br>100% had visible mould growth at some time in the study<br>92% of homes had condensation<br>40% of homes had water intrusion<br>67% of homes had musty odour<br><br>**Key findings:**<br>Occupants were not aware that their home had dampness problems with 60% of households removed mould growth on a regular basis but only

						23% of occupants considered their home to be damp (including structural dampness and condensation).<br><br>In this study, no significant associations were seen between spore concentration levels and that of air-conditioning, gas stove presence, damp house, blocked sub ventilation, cladding type, soil type, drainage, garden beds around the house or house age.
Built form and health<br>(Robertson, 1992)<br><br>**Data Year:**<br>< 1992	**Location:**<br>VIC<br>Melbourne<br><br>Climate zone 6<br><br>**Housing Types:**<br>Freestanding houses	Occupants with environmental sensitivities<br><br>Homes<br>N=40	QUAN<br>Prevalence study	**Risk factors:**<br>Regular exposure to a pollutants carried on airflow from bathrooms in homes with problems with dampness.<br><br>**Building characteristic risk factors:**<br>A combination of factors including; bathroom window orientation, site slope, number of window openings, reduced external/interval wind speeds.	PY	**Key findings with regards health impacts**:<br>Symptoms included from the housing occupants and/or visiting architect; hypersensitivity or allergy, bronchial complaints (sinus, cough, hay fever, including asthma), loss of sense of smell, skin complaints (eczema, itching, inflammation), fatigue and other (including headaches, hyperactivity, mood change).<br><br>**Key findings:**<br>Remedial measures may depend more upon wind characteristics than sun. Air flow may carry from damp room to bedrooms.
Allergic alveolitis due to wood-rot fungi<br>(Bryant & Rogers, 1991)<br><br>**Data Year:**<br>< 1991	**Location:**<br>NSW<br>Sydney<br><br>Climate zone 5<br><br>**Housing Types:**<br>Freestanding houses	Adults<br>N=12	QUAN<br>Case Control<br><br>HT, DS	**Risk factors:**<br>Indoor exposure to fungi from damp walls and dry-rot wood<br><br>**Building characteristic risk factors:**<br>Age of home >70 years (in <1991), poor housing condition, floor boards with extensive dry-wood damage, collapsing floor boards and mould growth in damp walls	Y*<br><br><br><br>Y*	**Key findings with regards health impacts**:<br>Domestic allergic alveolitis<br><br>**Biological findings:**<br>Elevated indoor fungi from damp walls and dry-rot wood
Theme – Building characteristics, indoor mould/mildew/fungi, housing biological data & allergens						
Predictors of high house dust mite allergen concentrations in residential homes in Sydney<br>(Mihrshahi et al., 2002)<br><br>**Data Year:**<br>(unsure, 1997 to 2001)	**Location:** NSW<br>Sydney<br><br>Climate zone 5/6<br><br>**Housing Types:**<br>Mixed	Pregnant women<br>N = 616	QUAN<br>Cross-sectional<br><br>IQ<br>DS	**Building characteristic risk factors:**<br>Age of home, wood foundation, weatherboard, beds with mattresses > 2 years old, woollen or synthetic blankets / quilts and carpeted floors as oppose to hard floors	Y**	**Biological findings:**<br>High house-dust-mite allergen (Der p 1) concentrations<br><br>**Prevalence:**<br>23.6% said yes to mould present in home (excluding mould in the bathroom)<br><br>**Key findings with regards to mould:**<br>Negative association - Visible mould growth did not have an independent effect on house-dust-mite allergen levels.

Residential characteristics influence Der p 1 levels in homes in Melbourne, Australia<br>(Dharmage, Bailey, Raven, Cheng, et al., 1999)<br><br>**Data Year:**<br>1996	**Location:**<br>VIC<br>Melbourne<br><br>Climate zone<br>6<br><br>**Housing Types:**<br>Freestanding houses	Young adults<br>N=485	QUAN<br>Cross-sectional<br><br>IQ, DS, RH	**Building characteristic risk factors:**<br>Observed dampness and visual mould<br>Houses built before 1980, with wooden floors built on stumps, with high relative humidity, with visible mould in the room, in beds with an old mattress or in beds without a quilt.	 Y*<br>Y*	**Biological findings:**<br>Predictors of Der p 1 levels in the bedrooms<br>Predictors of Der p 1 levels in the bedrooms<br><br>**Prevalence:**<br>36% homes had observed mould<br>26% homes had observed damp patches
Prevalence and residential determinants of fungi within homes in Melbourne, Australia.<br>(Dharmage, Bailey, Raven, Mitakakis, et al., 1999)<br><br>**Data Year:**<br>1996	**Location:**<br>VIC<br>Melbourne<br><br>Climate zone<br>6<br><br>**Housing Types:**<br>Freestanding houses	Young adults<br>N=485	QUAN<br>Cross-sectional<br><br>IQ, DS, IAS, VI	**Building characteristic risk factors:**<br>Season: Summer / Autumn<br>Visible mould on 2 or more surfaces<br><br>Damp patches / visible mould + no mechanical ventilation (celling fan in bedroom or kitchen exhaust fan)<br><br>Double brick walls, no solid fuel fire in last 12 months, no ceiling fan in bedroom, no kitchen exhaust fan, old carpets equal > 5 years old, infrequent natural ventilation, windows open, vacuuming > 1 week ago and presence of a cat or presence of a dog	 Y*<br>Y*<br><br>Y*<br><br>Y*	**Biological findings:**<br>High indoor fungal exposure (fungal propagules cfu/ m³)<br>High indoor fungal exposure (fungal propagules cfu/ m³)<br><br>High indoor fungal exposure (fungal propagules cfu/ m³)<br><br>High indoor fungal exposure (fungal propagules cfu/ m³)<br><br>**Prevalence:**<br>55% had viable fungal propagules concentrations > 500 cfu/ m³<br>36.5% had 1 or more surfaces with visible mould in the bedroom<br>25.6% had observed damp patches in either or both bedroom and adjoining room<br>90% had one or more mouldy surfaces had damp patches in both bedroom and the adjoining room.<br>27% of homes without damp patches in either the bedroom or adjoining room had one or more mouldy surfaces
Determinants of dust mite allergen concentrations in infant bedrooms in Tasmania<br>(Couper et al., 1998)<br><br>**Data Year:**<br>1995	**Location:**<br>TAS<br><br>Climate zone 7<br><br>**Housing Types:**<br>Freestanding houses	Infants<br>n=80<br>N=72	QUAN<br>Cross-sectional<br><br>SRQ, IQ<br>DS	**Building characteristic risk factors:**<br>Mould in the home (not in bathroom), low maximum temps in the infants bedroom, high indoor humidity and 6 or more residents<br><br>Frequent vacuuming (at least 1/ week) and carpeted bedrooms	 Y*<br><br>Y	**Biological findings:**<br>High house-dust-mite allergens (Der p1)<br><br>High house-dust-mite allergens (Der p1)<br><br>**Prevalence:**<br>34.3% had mould in bathroom in past month<br>20.8% yes to 'Have you noticed mould inside your house (excluding bathroom)?'

						18.8% had mould other than in bathroom in the past month<br>9.8% had mould in bedroom observed by the inspector
Indoor environmental factors associated with house-dust-mite allergen (Der p i) levels in south-eastern Australian houses<br>(M. H. Garrett, Hooper, & Hooper, 1998)<br><br>**Data Year:**<br>From 1994/95	**Location:**<br>VIC<br>Latrobe Valley<br><br>Climate zone 6<br><br>**Housing Types:**<br>Freestanding houses	Children<br>(n=148)<br>Homes<br>N=80	QUAN<br>Cross-sectional<br><br>SRQ, IQ, DS, IAS,	**Building characteristic risk factors:**<br>New houses (data from 1994/95) with brick cladding and slab-type foundations, wool carpets in bedrooms, presence of substantial visible mould growth, musty odour, wool bedding and inner spring mattresses and high indoor humidity RH > 50%	Y*	**Biological findings:**<br>High levels of house-dust-mite allergen<br><br>**Prevalence:**<br>29.3% bedrooms had observed substantial mould<br>70.7% bedrooms had observed none/slight mould<br>21.3% living rooms had observed substantial mould<br>78.8% living rooms had observed none/slight mould
Airborne Mould Levels and Related Environmental Factors in Australian Houses<br>(Godish et al., 1996)<br><br>**Data Year:**<br>1992	**Location:**<br>VIC<br>Latrobe Valley<br><br>Climate zone 6<br><br>**Housing Types:**<br>Freestanding houses	Homes<br>N=40	QUAN<br>Cross-sectional<br><br>IQ, IAS, RH<br>VI	**Building characteristic risk factors:**<br>Age of home >20 years (in 1992)<br>High indoor humidity RH equal > 70%<br>Water intrusion	 Y*<br>Y*<br>Y*	**Biological findings:**<br>Elevated indoor cultured mould levels<br>Elevated indoor cultured mould levels<br>Elevated indoor total mould spores<br><br>**Biological prevalence:**<br>58% of homes had one or more rooms exceeding 1,000 cfu/ m³<br>48% of homes had one or more rooms exceeding 10,000 cfu/ m³<br>100% of homes had detected Penicillium genus at significant higher concentration, in cultural mould samples, compared to the outdoor concentrations
Theme – Building characteristics & housing condition surveys (that report indoor mould/mildew/fungi)						
The Australian Housing Conditions Dataset: Technical Report<br>(Baker et al., 2018)<br><br>**Data Year:**<br>2016	**Location:**<br>NSW, VIC, SA<br><br>**Housing Types:**<br>Mixed	Adults<br>N=4501<br><br>Mixed tenure types	QUAN<br>Cross-sectional housing survey<br><br>SRQ, IQ	**Tenure/housing type breakdown:**<br>87.9% owner occupier<br>10.2% rented<br>83.7% separate house<br>9.2% semi-detached/terrace/townhouse<br>6.8% flat/apartment		**Prevalence poor housing conditions included:**<br>8.8% of dwellings had problems with mould<br>13.9% of dwellings had problems with rising damp<br>51.9% of dwellings had problems with cracks in walls/floors<br>16.9% of dwellings had problems of sinking/moving foundations<br>13.7% of dwellings had problems with roof defects<br>**Prevalence housing quality:**<br>1.1% had self-assessed "Poor" quality of dwelling<br>0.3% had self-assessed "Very poor" quality of dwelling
1994 - Australian Housing Survey - Housing Characteristics Costs Conditions. ABS 41820<br>(Australian Bureau of Statistics, 1996)<br><br>**Data Year:**<br>1994	**Location:**<br>National<br><br>**Housing Types:**<br>Mixed	Homes<br>N=14,456<br><br>Mixed tenure types	QUAN<br>Cross-sectional housing survey<br><br>SRQ, IQ	**Building characteristic risk factors**:<br>Age of dwelling<br>Homes 10 years or less in 1994<br>Homes 10 years or more in 1994<br>Problems arising from roofs (tiles or metal sheeting)<br>Problems arising from outer walls (double brick, brick veneer, timber)	 <br>Y<br>Y<br>Y<br><br>Y	**Prevalence poor housing conditions included:**<br>9.54% homes had mould or mildew<br>20.22% homes had mould or mildew<br>17.97% of total homes had mould or mildew<br>Mould / mildew / water penetration - 8% of dwellings reported leaking roof or ceiling<br>Water penetration

				Problems arising from leaking window frames (aluminium or timber)	Y	Water penetration
Theme – Building characteristics, conditions, defects & 'root cause' studies						
Mould risk evaluations in residential buildings via site audits and longitudinal monitoring (Kempton et al., 2021)<br><br>**Data Year:**<br>(2016 to 2017 remediations)<br>2017, 2018 to 2019	**Location:**<br>Sydney<br><br>Climate zone 5/6<br><br>**Housing Types:**<br>Mixed	N=233<br>N=30 subset<br>N=5 subset	QUAN<br>Case Series<br>Houses	**Building characteristic risk factors:**<br>Bedroom RH >80%<br>High wall surface temperatures compared to the room air dewpoint temperature.<br><br>**Root cause of mould from N=233 property insurance reports:**<br>70.8% Ventilation<br>39.1% Leak identified<br>19.3% Only a leak<br>15% Lack of natural light<br>7.3% Rising damp<br>1.7% External drainage issues	 Y<br>Y	**Risk for:**<br>Mould growth<br>Mould growth<br><br>**Prevalence mould growth from insurance claims:**<br>Average number of rooms affected in claims were 2<br>78% Bathrooms were affected by<br>35% at least 1 bedroom affected<br>32% kitchens<br>24% lounge room<br><br>**Key findings:**<br>86.7% of remediation works reported painting - which included painting over surfaces that was affected by discolouration by mould. 40% of the subset remediated homes (N=30) experienced mould regrowth within 12 months. Remediation without addressing the "root cause" of mould issues is not effective in preventing mould growth from reoccurring in the short term. A number of mould growth modelling tools were unable to reliably predict the risk of mould occurrence in the N=5 monitored properties.
An examination of building defects in residential multi-owned properties (N. Johnston & Reid, 2019)<br><br>**Data Year:**<br>2003 to 2018	**Location:**<br>NSW, Qld, VIC<br><br>**Housing Types:**<br>Medium / high density housing / apartments / units	N=212<br>Building defect audit reports	MMR<br><br>Defect audit reports<br><br>QUALi	**Building characteristic risk factors:**<br>Lack of adequate ventilation causing condensation and mould<br><br>**Prevalence of water/ingress/moisture related defects identified:**<br>29% from all construction systems defects<br>71% from waterproofing defects<br>56% from roofing and rainwater disposal<br>33% from defects in building fabric / cladding<br>9% from structural defects		**Key findings by expert opinions:**<br>Most common defects noted in the reports were, water damage and mould to plasterboard<br><br>Mould that has arisen due to water ingress defects is often present and has the potential to lead to serious health implications for residents. The lack of care by trades in properly managing mould often leads to spores embedding or remaining in lots. Ineffective mould remediation by trades leading to spread of spores to other areas, spores embedding or remaining in lots. Industry concerns about physical health impacts. Concerns about occupants living in apartments affected by mould.

Cracks in the Compact City: Tackling defects in multi-unit strata housing Final Project Report (Crommelin et al., 2021)<br><br>**Data Year:**<br>2008 to 2017	**Location:**<br>Sydney<br><br>Climate zone 5/6<br><br>**Housing Types:**<br>Apartments / units	N=635	MMR<br><br>Secondary data, reports,<br><br>QUALi	**Water related defects:**<br>Water leak / penetration / ingress - Wall, Slab<br>Moisture / Mould / Humidity / Dampness<br>Water pond / Water flooding<br>Waterproofing defect<br>Drainage defects<br>Pipes<br>Blocked weephole<br>Shower booth, Basin etc.<br>Tap	 Y<br>Y<br>Y<br>Y<br>Y<br>Y<br>Y<br>Y<br>Y	**Prevalence of type of water related defects identified:**<br>18% of defects identified<br>6% of defects identified<br>6% of defects identified<br>5% of defects identified<br>5% of defects identified<br>4% of defects identified<br>2% of defects identified<br>1% of defects identified<br>1% of defects identified
Scoping Study of Condensation in Residential Buildings (Dewsbury, Law, Potgieter, et al., 2016)<br><br>**Data Year:**<br>2015 to 2016	**Location:**<br>National<br><br>**Housing Types:**<br>Mixed	Expert opinions n= 2662	MMR<br><br>QUALi	**Building characteristic risk factors**:<br>Newer building typologies may not be able to manage vapour pressure, condensation and mould. Many interrelated factors including design, construction and building occupation.<br><br>**The top three potential causes of excess condensation**:<br>1) the occupant being unaware of their behaviours contributing to condensation;<br>2) an increase in air-tighter buildings and sealing of windows and doors; and<br>3) the occupant reluctant to open windows and doors due to energy costs.	PY	**Key findings by expert opinions:**<br>Potential for surface/interstitial condensation leading to visible mould growth and degradation of building materials for new code compliant homes<br><br>**Prevalence:**<br>There is a national concern about condensation in buildings constructed since 2004 may include 40% of all Class 1 and Class 2 buildings.
The Unintended Consequence of Building Sustainably in Australia (Law & Dewsbury, 2018)<br><br>**Data Year:**<br>2014	**Location:**<br>TAS<br><br>Climate zone 7<br><br>**Housing Types:**<br>Freestanding houses	Homes N=2	QUAN Case Series Houses<br><br>RH<br>IAS<br>DS	**Building characteristic risk factors**:<br>Code compliant homes in a cool climate.<br>Thermal bridging - metal window frames, around an uninsulated recessed ceiling light fitting, condensation underside roof. External walls adjoining unheated spaces. Poorly installed insulation. Vapour impermeable vinyl floor finish. Unventilated external wall cavities. Non breathable wall wraps/foil wraps.	Y	**Key findings by expert opinions:**<br>Surface/interstitial condensation leading to visible mould growth
Investigation of Destructive Condensation in Australian Cool temperate Buildings Appendix 3: Case Study House 1, 2 & 3 (Dewsbury, Law, & Henderson, 2016c, 2016d, 2016a, 2016b)	**Location:**<br>TAS<br>Semi-rural<br><br>Climate zone 7	Homes N=3	QUAN Case Series Houses<br><br>RH<br>IAS	**Building characteristic risk factors**:<br>Code compliant homes in a cool climate.<br>Thermal bridging - condensation on/around metal window frames, insulation in direct contact with roof sarking system, around an uninsulated recessed ceiling light fitting,	Y	**Key findings by expert opinions:**<br>Surface/interstitial condensation leading to visible mould growth<br><br>**Root cause of mould from:**<br>Exposed building envelope<br>Condensation underside attic/roof

**Data Year:**<br>2014/15	**Housing Types:**<br>Freestanding houses		DS	condensation underside roof, external walls adjoining unheated spaces.		Ground moisture in subfloor area<br>Poor ground drainage leading<br>Inappropriate site drainage.<br>Inappropriate installation of roof vents<br>Incorrect installation of foil and insulation sarking system<br>Unprotected wall penetrations<br>Unprotected external timber structure<br>Poorly installed insulation<br>Vapour impermeable floor finish<br>Unventilated external wall cavities<br>Non breathable wall wraps/foil wraps<br>Poor quality workmanship / construction
Theme – Energy retrofit, mould/mildew/fungi intervention studies						
Addressing health and equity in residential low carbon transitions– Insights from a pragmatic retrofit evaluation in Australia (Willand et al., 2019)<br><br>**Data Year:**<br>2014/15	**Location:**<br>VIC<br><br>Climate zone 6<br><br>**Housing Types:**<br>Mixed	Low-income older households N=30	MMR Before/after studies<br><br>QUALi VI, IAS	**Building characteristic risk factors:**<br>Poorly ventilated areas behind curtains<br>Cold surfaces of single glazed windows<br><br>**Protective occupant behaviours:**<br>Occupant behaviours that help avoid mould.	Y	**Biological findings:**<br>Prevalence of mould<br><br>**Key findings:**<br>Approx. 50% of occupants, kept windows or doors permanently open in the house to accommodate dogs, avoid mould, improve breathing, mitigate feelings of claustrophobia or due to having grown up sleeping on enclosed verandas or "sleep-outs"
Indoor Air Quality problems as a result of installing split system HVAC units in mass housing accommodations (Midson et al., 2012)<br><br>**Data Year:**<br><2012	**Location:**<br>WA<br>North-west<br>Remote<br><br>Climate zone unknown<br><br>**Housing Types:**<br>Mass accom. blocks	N=12 rooms/areas	QUAN Intervention study	**Building characteristic risk factors**:<br>Split system air-conditioning units	Y	**Biological impact:**<br>Source of mould growth in mould affected properties.
Dishwashers - A man-made ecological niche accommodating human opportunistic fungal pathogens (Zalar et al., 2011)<br><br>**Data Year:**<br><2010	**Location:**<br>International study including VIC<br><br>Climate zone 6	N=189 dishwashers (N=7 Australian households)	QUAN Prevalence study	**Building characteristic risk factors:** Plastic seals in the dishwasher door.	Y*	**Biological findings:**<br>62% of dishwashers were positive for fungi, and 56% of these accommodated pathogenic fungi responsible for causing systemic disease in humans and frequently colonize lungs of patients with cystic fibrosis.

Hot water extraction in carpeted homes in Western Australia: Effect on airborne fungal spora<br>(C. Cheong et al., 2007)<br><br>**Data Year:**<br>< 2007	**Location:**<br>WA<br>Perth<br><br>Climate Zone 5<br><br>**Housing Types:**<br>Freestanding houses	N=14<br>Single family dwellings	QUAN<br>Case Control<br><br>IAS, VI, DS	**Intervention:**<br>Professional cleaning domestic carpet with 'wet extraction' method.<br><br>**Building characteristic risk factors:**<br>Carpets without professional cleaning interventions, in this study 'wet extraction', or have poor cleaning maintenance.	 Y<br><br><br><br>Y	**Biological impact:**<br>Increased quantities and variety of fungal and airborne particulate loads available for resuspension.<br><br><br>Higher levels of fungi
Intervention study of airborne fungal spora in homes with portable HEPA filtration units<br>(C. D. Cheong et al., 2004)<br><br>**Data Year:**<br>2000	**Location:**<br>WA<br>Perth<br><br>Climate Zone 5<br><br>**Housing Types:**<br>Freestanding houses	N=10<br>Single family dwellings	QUAN<br>Case Control<br><br>IAS, VI, DS	**Intervention:**<br>Homes fitted with portable HEPA filtration units.	 Y	**Biological impact:**<br>Overall average reduction of 35% in airborne viable indoor fungal levels and a 38% reduction in airborne fine particles.

Notes: PY = Perceived association, Y = Positive association, Y* = Significant association (P<0.05), Y** = Significant association (P<0.001), SRQ = Self-reported questionnaire, IQ = Interviewer questionnaire, QUALi = Qualitative interviews QUALo + Open ended questions, VS = Visual storytelling, VI=Visual inspection, IAS = Indoor air sampling, DS = Dust sampling, HT = Human tests, RH=Relative humidity, n=people, N=homes, Mixed = Unit / townhouse / freestanding house.
Grey background indicates that the study was not published in an academic journal/publication.

References

Andersen, M.J.; Skinner, A.; Williamson, A.B.; Fernando, P.; Wright, D. Housing conditions associated with recurrent gastrointestinal infection in urban aboriginal children in NSW, Australia: Findings from SEARCH. *Aust. N. Z. J. Public Health* **2018**, *42*, 247–253. https://doi.org/10.1111/1753-6405.12786.

Andersen, M.J.; Williamson, A.B.; Fernando, P.; Redman, S.; Vincent, F. "There's a housing crisis going on in Sydney for aboriginal people": Focus group accounts of housing and perceived associations with health. *BMC Public Health* **2016**, *16*, 429. https://doi.org/10.1186/s12889-016-3049-2.

Andersen, M.J.; Williamson, A.B.; Fernando, P.; Wright, D.; Redman, S. Housing conditions of urban households with aboriginal children in NSW Australia: Tenure type matters. *BMC Public Health* **2018**, *18*, 70. https://doi.org/10.1186/s12889-017-4607-y.

Australian Bureau of Statistics. *4182.0—Housing characteristics, costs and conditions, Australia, 1994*; Commonwealth of Australia, 1996; pp. 1–66. Available online: https://www.abs.gov.au/AUSSTATS/abs@.nsf/DetailsPage/4182.01994?OpenDocument (accessed 1 November 2021).

Baker, E.; Daniel, L. Rental Insights: A COVID-19 Collection. Available online: https://www.ahuri.edu.au/sites/default/files/migration/documents/Rental-Insights-A-COVID-19-Collection.pdf (accessed on 4 November 2021).

Baker, E.; Daniel, L.; Bentley, R.; Pawson, H.; Stone, W.; Rajagopalan, P.; Hulse, K.; Beer, A.; London, K.; Zillante, G.; et al. *The Australian Housing Conditions Dataset: Technical Report*; The University of Adelaide—Healthy Cities Research: Adelaide, SA, Australia, 2018; pp. 1–28. Available online: https://dataverse.ada.edu.au/file.xhtml?fileId=9876&version=1.0 (accessed on 31 January 2022).

Bower, M.; Buckle, C.; Rugel, E.; Donohoe-Bales, A.; McGrath, L.; Gournay, K.; Barrett, E.; Phibbs, P.; Teesson, M. 'Trapped', 'anxious' and 'traumatised': COVID-19 intensified the impact of housing inequality on Australians' mental health. *Int. J. Hous. Policy* **2021**, 1–32. https://doi.org/10.1080/19491247.2021.1940686.

Bryant, D.H.; Rogers, P. Allergic alveolitis due to wood-rot fungi. *Allergy Proc.* **1991**, *12*, 89–94. https://doi.org/10.2500/108854191779011855.

Cheong, C.D.; Neumeister-Kemp, H.G.; Dingle, P.W.; Hardy, G.S.J. Intervention study of airborne fungal spora in homes with portable HEPA filtration units. *J. Environ. Monit.* **2004**, *6*, 866–873. https://doi.org/10.1039/b408135h.

Cheong, C.; Neumeister-Kemp, H.G.; Kemp, P.C. Hot water extraction in carpeted homes in western Australia: Effect on airborne fungal spora. In Proceedings of the 14th International Union of Air Pollution Prevention and Environmental Protection Associations (IUAPPA) World Congress 2007, 18th Clean Air Society of Australia and New Zealand (CASANZ) Conference, Brisbane, QLD, Australia, 9–13 September 2007.

Choice; National Shelter. Unsettled: Life in Australia's Private Rental Market. 2016. Available online: http://shelter.org.au/site/wp-content/uploads/The-Australian-Rental-Market-Report-Final-Web.pdf (accessed 19 November 2021).

Choice; National Shelter; The National Assoication of Tenant Organisations (NATO); Martin, C. Disrupted: The Consumer Experience of Renting in Australia. Available online: https://www.shelterwa.org.au/wp-content/uploads/2019/08/Disrupted-2018ReportbyCHOICENationalShelterandNATO.pdf (accessed on 29 October 2021).

Commonwealth of Australia. House of representatives standing committee on health aged care and sport. In *Report on the Inquiry into Biotoxin-Related Illnesses in Australia*; Parliament of Australia: Canberra, Australia, 2018. Available online: https://parlinfo.aph.gov.au/parlInfo/download/committees/reportrep/024194/toc_pdf/ReportontheInquiryintoBiotoxin-relatedIllnessesinAustralia.pdf;fileType=application%2Fpdf (accessed on 16 January 2022).

Couper, D.; Ponsonby, A.L.; Dwyer, T. Determinants of dust mite allergen concentrations in infant bedrooms in Tasmania. *Clin. Exp. Allergy* **1998**, *28*, 715–723. https://doi.org/10.1046/j.1365-2222.1998.00307.x.

Crommelin, L.; Thompson, S.; Easthope, H.; Loosemore, M.; Yang, H.; Buckle, C.; Randolph, B. *Cracks in the Compact City: Tackling Defects in Multi-Unit Strata Housing*; Final Project Report; 2021. Sydney, Australia. Available online: https://cityfutures.ada.unsw.edu.au//research/projects/defects-strata/ (accessed on 1 November 2021).

Dewsbury, M.; Law, T.; Henderson, A.D. Investigation of Destructive Condensation in Australian Cool Temperate Buildings Appendix 1: Case Study House 1. Available online: https://www.researchgate.net/publication/301895039_Investigation_of_destructive_condensation_in_Australian_cool-temperate_buildings_Appendix_1 (accessed on 31 January 2022).

Dewsbury, M.; Law, T.; Henderson, A.D. Investigation of Destructive Condensation in Australian Cool Temperate Buildings Appendix 2: Case Study House 2. Available online: https://www.researchgate.net/publication/301894762_Investigation_of_destructive_condensation_in_Australian_cool-temperate_buildings_Appendix_2 (accessed on 31 January 2022).

Dewsbury, M.; Law, T.; Henderson, A.D. Investigation of Destructive Condensation in Australian Cool Temperate Buildings Appendix 3: Case Study House 3. Available online: https://www.researchgate.net/publication/301894764_Investigation_of_destructive_condensation_in_Australian_cool-temperate_buildings_Appendix_3 (accessed on 31 January 2022).

Dewsbury, M.; Law, T.; Henderson, A.D. Investigation of Destructive Condensation in Australian Cool-Temperate Buildings: Final Report. Available online: https://www.researchgate.net/publication/301894751_Final_report_-_Investigation_of_destructive_condensation_in_Australian_cool-temperate_buildings (accessed on 16 January 2022).

Dewsbury, M.; Law, T.; Potgieter, J.; Fitz-Gerald, D.; McComish, B.; Chandler, T.; Soudan, A. Scoping Study of Condensation in Residential Buildings: Final Report (23 September 2016) (for the Australian Building Codes Board). 2016. Available online: https://www.abcb.gov.au/sites/default/files/resources/2020/Scoping_Study_of_Condensation_in_Residential_Buildings.pdf (accessed on 5 November 2021).

Dharmage, S.; Bailey, M.; Raven, J.; Abeyawickrama, K.; Cao, D.; Guest, D.; Rolland, J.; Forbes, A.; Thien, F.; Abramson, M.; et al. Mouldy houses influence symptoms of asthma among atopic individuals. *Clin. Exp. Allergy* **2002**, *32*, 714–720. https://doi.org/10.1046/j.1365-2222.2002.01371.x.

Dharmage, S.; Bailey, M.; Raven, J.; Cheng, A.; Rolland, J.; Thien, F.; Forbes, A.; Abramson, M.; Walters, E.H. Residential characteristics influence der p 1 levels in homes in Melbourne, Australia. *Clin. Exp. Allergy* **1999**, *29*, 461–469. https://doi.org/10.1046/j.1365-2222.1999.00513.x.

Dharmage, S.; Bailey, M.; Raven, J.; Mitakakis, T.; Cheng, A.; Guest, D.; Rolland, J.; Forbes, A.; Thien, F.; Abramson, M.; et al. Current indoor allergen levels of fungi and cats, but not house dust mites, influence allergy and asthma in adults with high dust mite exposure. *Am. J. Respir. Crit. Care Med*. **2001**, *164*, 65–71. https://doi.org/10.1164/ajrccm.164.1.9911066.

Dharmage, S.; Bailey, M.; Raven, J.; Mitakakis, T.; Thien, F.; Forbes, A.; Guest, D.; Abramson, M.; Walters, E.H. Prevalence and residential determinants of fungi within homes in Melbourne, Australia. *Clin. Exp. Allergy* **1999**, *29*, 1481–1489. https://doi.org/10.1046/j.1365-2222.1999.00640.x.

Garrett, M.H.; Hooper, B.M.; Hooper, M.A. Indoor environmental factors associated with house-dust-mite allergen (der p 1) levels in south-eastern Australian houses. *Allergy* **1998**, *53*, 1060–1065. https://doi.org/10.1111/j.1398-9995.1998.tb03815.x.

Garrett, M.H.; Rayment, P.R.; Hooper, M.A.; Abramson, M.J.; Hooper, B.M. Indoor airborne fungal spores, house dampness and associations with environmental factors and respiratory health in children. *Clin. Exp. Allergy* **1998**, *28*, 459–467. https://doi.org/10.1046/j.1365-2222.1998.00255.x.

Godish, D.; Godish, T.; Hooper, B.; Hooper, M.; Cole, M. Airborne mould levels and related environmental factors in Australian houses. *Indoor Built Environ.* **1996**, *5*, 148–154. https://doi.org/10.1177/1420326X9600500305.

Haddad, S.; Pignatta, G.; Paolini, R.; Synnefa, A.; Santamouris, M. An extensive study on the relationship between energy use, indoor thermal comfort, and health in social housing: The case of the New South Wales, Australia. In *Proceedings of the IOP Conference Series: Materials Science and Engineering, Bari, Italy, 1 September 2019*; Institute of Physics Publishing: Bristol, UK, 2019; Volume 609, p. 042067.

Hall, K.K.; Chang, A.B.; Anderson, J.; Arnold, D.; Goyal, V.; Dunbar, M.; Otim, M.; O'Grady, K.-A.F. The incidence and short-term outcomes of acute respiratory illness with cough in children from a socioeconomically disadvantaged urban community in Australia: A community-based prospective cohort study. *Front. Pediatr.* **2017**, *5*, 228. https://doi.org/10.3389/fped.2017.00228.

Johnston, N.; Reid, S. An Examination of Building Defects in Residential Multi-Owned Properties. Available online: https://www.griffith.edu.au/__data/assets/pdf_file/0022/831217/Examining-Building-Defects-Research-Report-S-Reid-N-Johnston.pdf (accessed on 1 November 2021).

Johnston, S.C.; Staines, D.R.; Marshall-Gradisnik, S.M. Epidemiological characteristics of chronic fatigue syndrome/myalgic encephalomyelitis in Australian patients. *Clin. Epidemiol*. **2016**, *8*, 97–107. https://doi.org/10.2147/CLEP.S96797.

Kempton, L.; Kokogiannakis, G.; Cooper, P. Mould risk evaluations in residential buildings via site audits and longitudinal monitoring. *Build. Environ.* **2021**, *191*, 107584. https://doi.org/10.1016/j.buildenv.2020.107584.

Law, T.; Dewsbury, M. The unintended consequence of building sustainably in Australia. In *Sustainable Development Research in the Asia-Pacific Region. World Sustainability Series*; Leal, F.W., Rogers, J., Iyer-Raniga, U., Eds.; Springer: Cham, Switzerland, 2018; pp. 525–547. ISBN 978-3-319-73293-0.

Martin, S. ANRES: A Snapshot of Living with Environmental Sensitivities in Australia in 2019. Available online: https://anres.org/2019-anres-data-update/ (accessed on 5 November 2021).

Matheson, M.; Abramson, M.J.; Dharmage, S.C.; Forbes, A.B.; Raven, J.M.; Thien, F.C.K.; Walters, E.H. Changes in indoor allergen and fungal levels predict changes in asthma activity among young adults. *Clin. Exp. Allergy* **2005**, *35*, 907–913. https://doi.org/10.1111/j.1365-2222.2005.02272.x.

Mészáros, D.; Burgess, J.; Walters, E.H.; Johns, D.; Markos, J.; Giles, G.; Hopper, J.; Abramson, M.; Dharmage, S.C.; Matheson, M. Domestic airborne pollutants and asthma and respiratory symptoms in middle age. *Respirology* **2014**, *19*, 411–418. https://doi.org/10.1111/resp.12245.

Midson, W.; Cheong, C.; Neumeister-Kemp, H.; White, K. Indoor air quality problems as a result of installing split system HVAC units in mass housing accommodations. In Proceedings of the 10th International Healthy Buildings Conference, Brisbane, QLD, Australia, July 2012.

Mihrshahi, S.; Marks, G.; Vanlaar, C.; Tovey, E.; Peat, J. Predictors of high house dust mite allergen concentrations in residential homes in Sydney. *Allergy* **2002**, *57*, 137–142. https://doi.org/10.1034/j.1398-9995.2002.5720999.x.

Ponsonby, A.-L.; Couper, D.; Dwyer, T.; Carmichael, A.; Kemp, A.; Cochrane, J. The relation between infant indoor environment and subsequent asthma. *Epidemiology* **2000**, *11*, 128–135. https://doi.org/10.1097/00001648-200003000-00008.

Robertson, H.J. Built form and health. *Indoor Environ*. **1992**, *1*, 238–250. https://doi.org/10.1177/1420326X9200100408.

Robertson, H.J. Spatial characteristics of southeast australian housing linked with allergic complaint. *Build. Environ.* **2001**, *36*, 931–937. https://doi.org/10.1016/S0360-1323(00)00050-0.

Saltos, N.; Saunders, N.A.; Bhagwandeen, S.B.; Jarvie, B. Hypersensitivity pneumonitis in a mouldy house. *Med. J. Aust.* **1982**, *2*, 244–246. https://onlinelibrary.wiley.com/doi/abs/10.5694/j.1326-5377.1982.tb124354.x

Trewin, D. Australian Housing Survey—Housing Characteristics, Costs and Conditions, Cat No. 4182.0; 1999. Available online: https://www.ausstats.abs.gov.au/ausstats/subscriber.nsf/0/D9B696BEE455C74CCA2569890002D180/$File/41820_1999.pdf (accessed on 16 January 2022).

Tyndall, J. AACODS Checklist for Appraising Grey Literature. Available online: https://dspace.flinders.edu.au/xmlui/bitstream/handle/2328/3326/AACODS_Checklist.pdf;jsessionid=A9241274C90700E1A8E77D8534BC8788?sequence=4 (accessed on 20 November 2021).

Victorian Council of Social Service (VCOSS). Decent Not Dodgy. “Secret Shopper” Survey. Available online: https://apo.org.au/sites/default/files/resource-files/2010-07/apo-nid22132.pdf (accessed on 16 January 2022).

Willand, N.; Maller, C.; Ridley, I. Addressing health and equity in residential low carbon transitions—Insights from a pragmatic retrofit evaluation in Australia. *Energy Res. Soc. Sci*. **2019**, *53*, 68–84. https://doi.org/10.1016/j.erss.2019.02.017.

Zalar, P.; Novak, M.; de Hoog, G.S.; Gunde-Cimerman, N. Dishwashers—A man-made ecological niche accommodating human opportunistic fungal pathogens. *Fungal Biol*. **2011**, *115*, 997–1007. https://doi.org/10.1016/j.funbio.2011.04.007.

Zhang, G.; Spickett, J.; Lee, A.H.; Rumchev, K.; Stick, S. Household hygiene practices in relation to dampness at home and current wheezing and rhino-conjunctivitis among school age children. *Pediatr. Allergy Immunol*. **2005**, *16*, 587–592. https://doi.org/10.1111/j.1399-3038.2005.00325.x.

Ziersch, A.; Due, C.; Walsh, M.; Arthurson, K. *Belonging Begins at Home: Housing, Social Inclusion and Health and Wellbeing for People from Refugee and Asylum Seeking Backgrounds*; Flinders Press: Bedford Park, SA, Australia, 2017. ISBN 9780648146018. https://researchnow.flinders.edu.au/en/publications/belonging-begins-at-home-housing-social-inclusion-and-health-and-

Ziersch, A.; Walsh, M.; Due, C.; Duivesteyn, E. Exploring the relationship between housing and health for refugees and asylum seekers in south Australia: A qualitative study. *Int. J. Environ. Res. Public Health* **2017**, *14*, 1036. https://doi.org/10.3390/ijerph14091036.
